# Deep learning-enabled analysis reveals distinct neuronal phenotypes induced by aging and cold-shock

**DOI:** 10.1186/s12915-020-00861-w

**Published:** 2020-09-23

**Authors:** Sahand Saberi-Bosari, Kevin B. Flores, Adriana San-Miguel

**Affiliations:** 1 Department of Chemical and Biomolecular Engineering, North Carolina State University, Raleigh, NC 27695 USA; 2 Department of Mathematics, North Carolina State University, Raleigh, NC 27695 USA

**Keywords:** Deep learning, Convolutional neural networks, Neurodegeneration, Neuronal beading, Aging, Machine learning, Phenotyping, *C. elegans*

## Abstract

**Background:**

Access to quantitative information is crucial to obtain a deeper understanding of biological systems. In addition to being low-throughput, traditional image-based analysis is mostly limited to error-prone qualitative or semi-quantitative assessment of phenotypes, particularly for complex subcellular morphologies. The PVD neuron in *Caenorhabditis elegans*, which is responsible for harsh touch and thermosensation, undergoes structural degeneration as nematodes age characterized by the appearance of dendritic protrusions. Analysis of these neurodegenerative patterns is labor-intensive and limited to qualitative assessment.

**Results:**

In this work, we apply deep learning to perform quantitative image-based analysis of complex neurodegeneration patterns exhibited by the PVD neuron in *C. elegans*. We apply a convolutional neural network algorithm (Mask R-CNN) to identify neurodegenerative subcellular protrusions that appear after cold-shock or as a result of aging. A multiparametric phenotypic profile captures the unique morphological changes induced by each perturbation. We identify that acute cold-shock-induced neurodegeneration is reversible and depends on rearing temperature and, importantly, that aging and cold-shock induce distinct neuronal beading patterns.

**Conclusion:**

The results of this work indicate that implementing deep learning for challenging image segmentation of PVD neurodegeneration enables quantitatively tracking subtle morphological changes in an unbiased manner. This analysis revealed that distinct patterns of morphological alteration are induced by aging and cold-shock, suggesting different mechanisms at play. This approach can be used to identify the molecular components involved in orchestrating neurodegeneration and to characterize the effect of other stressors on PVD degeneration.

## Background

**Fig. 1:**
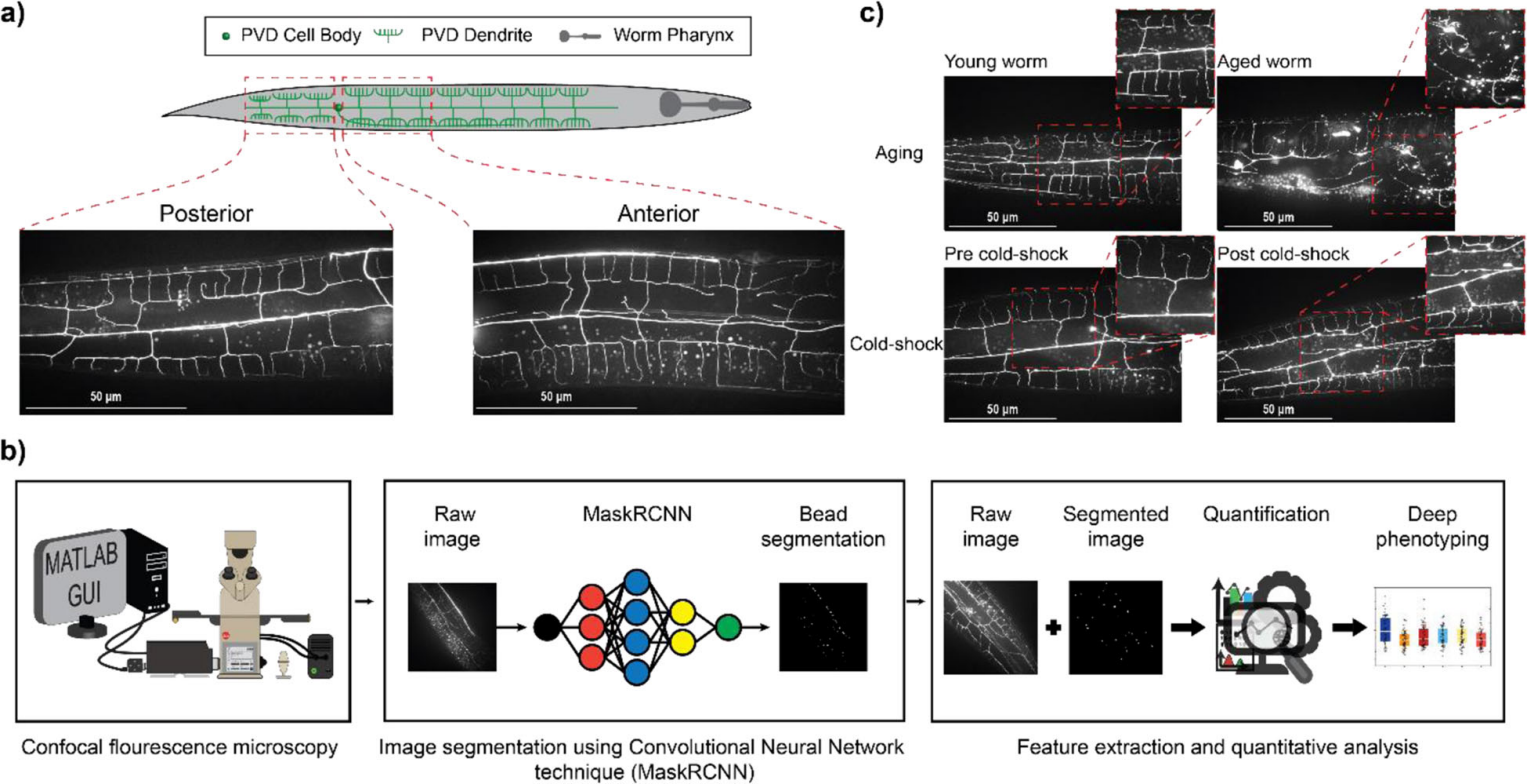
Quantitative analysis of PVD neurodegeneration by deep learning. **a** Schematic of PVD neuron with menorah-like dendritic branches. Fluorescence images of PVD anterior and posterior to the cell body. **b** Schematic of quantitative analysis pipeline to study PVD neurodegeneration. **c** Aging and acute cold-shock induce neurodegeneration on PVD dendrites. These two stressors increase the formation of bubble-like protrusions along the dendritic arbors of PVD

Aging, environmental stressors, and injury can induce reversible or irreversible changes at the subcellular, cellular, and tissue levels of an organism [1–11]. The *Caenorhabditis elegans* nervous system is not an exception and undergoes morphological and functional deterioration under these conditions. Morphological phenotypes indicative of neurodegeneration in this roundworm include somatic outgrowth, distorted soma, branched and wavy dendrites, and dendritic beading [2, 8, 12–18]. For instance, degenerative axonal beading has been observed and identified previously in various neurons such as ALM, PLM, and HSN [8, 19–22], and in dopaminergic neurons upon exposure to genotoxins [23]. The ability of neurons to recover from degeneration has also been studied. For instance, Oren-Suissa et al. found that primary dendrites in the PVD neuron reconnect via branch fusion following laser surgery [24]. PVD is a widely studied multidendritic nociceptor neuron that responds to harsh touch (mechanosensor) and cold temperatures (thermosensor) (Fig. 1a) [25–32]. Prior work has identified genetic pathways important for organization of dendritic branches and dendritic self-avoidance [33–38]. Dendritic organization in PVD is also affected by aging; while young animals have well-organized menorah-like dendritic structures, these tend to be replaced by non-uniform and chaotic outgrowth of dendritic branches [37]. Recently, Lezi et al. identified the formation of protrusions (or beading) along the dendrites of PVD during aging, through a process driven by the expression of an antimicrobial peptide [39]. They also identified a decrease in nematode’s responsiveness to harsh touch as nematodes age coinciding with the increase in number of bubble-like protrusions throughout the dendrite. Furthermore, mutants with delayed bead formation also exhibit a delayed emergence of harsh touch defects [39]. While these correlations suggest beading accompanies aging, their functional outcomes are still to be determined.

Characterization of PVD beading has thus far been performed by visual inspection and manual counting of fluorescent images, which is labor-intensive and time-consuming and does not provide additional information about the observed morphological changes, aside from number of beads. Traditional image processing approaches typically rely on intensity difference for image segmentation [40–42]. The protrusions that appear in PVD have fluorescence intensities similar to the rest of neuron and autofluorescent lipid droplets. Thus, traditional image processing approaches are unable to perform the challenging segmentation of PVD protrusions. Quantitative analysis of PVD neurodegeneration morphology is important to understand the root causes of neurodegeneration. Machine learning has proven useful for analysis of biological systems and deep phenotyping [9, 43–45]. Specifically, deep phenotyping and machine learning have been implemented in several *C. elegans* studies. San-Miguel et al. implemented a deep phenotyping pipeline to study synaptic patterning in the DA9 motoneuron [43]. Hakim et al. developed a platform called WorMachine which is comprised of image processing, deep learning, and machine learning techniques to perform assays such as supervised classification of binary-sex phenotype, scoring continuous-sexual phenotypes, quantifying the effects of two different RNA interference treatments, and measuring intracellular protein aggregation [46]. Kaltdorf et al. combined a machine learning technique with an image segmentation workflow to develop an automated method to classify Clear Core (CCV) and Dense Core (DCV) synaptic vesicles [47]. Wang et al. investigated cell movement by integrating deep reinforcement learning embedded in a modeling system to analyze 3D time-lapse microscopy images [48]. Wu et al. imaged neuronal activity of *C. elegans* in 3D by training a deep neural network to refocus two-dimensional fluorescence images into user-defined three-dimensional surfaces [49]. In this work, we sought to integrate cutting-edge deep learning approaches to segment beads in PVD fluorescence images from live animals (Fig. 1b). In this pipeline, confocal fluorescence images are fed to the trained Mask R-CNN-based algorithm for segmentation. The masks obtained are then used, along with its corresponding original image, to extract quantitative metrics describing the morphological changes of PVD, some of which include intensity-based information. Convolutional neural networks (CNNs) have recently shown state-of-the-art performance in image segmentation tasks across a wide range of biological and biomedical image datasets [50–56]. Here, we utilize Mask R-CNN [57], a CNN model that is designed to predict binary instance masks (one mask per predicted bead object) from an image to detect PVD beads. We follow this user-free segmentation approach with multiparametric phenotyping of PVD by extracting 46 quantitative features that describe beading patterns. These metrics include number of beads, cumulative area occupied by beads, average bead size, and average pairwise inter-bead distance. We take advantage of the quantitative data provided by this pipeline to track subtle neurodegenerative phenotypes caused by different physiological stressors (Fig. 1c). We validate our pipeline by assessing the effects of aging on PVD beading, and recapitulate previously observed changes [39]. In addition, we identify a previously unknown degenerative effect of exposure to acute cold-shock on neuronal structure. Finally, we show that this deep phenotyping approach enables predicting the biological status of a nematode (young, aged, cold-shocked) based on the quantitative metrics generated by the pipeline with over 85% accuracy. This analysis reveals that different stressors (aging and cold-shock) induce distinct neurodegenerative phenotypes hinting at potentially different underlying neurodegeneration mechanisms. This approach enabled automating image analysis of PVD neurodegeneration thus increasing throughput, eliminating the human bias and error introduced by manual assessment, and facilitated high-content quantification of the subtle neurodegenerative changes in PVD, unfeasible in conventional methods.

## Results and discussion

### Training the Mask R-CNN algorithm to perform complex image segmentation

**Fig. 2:**
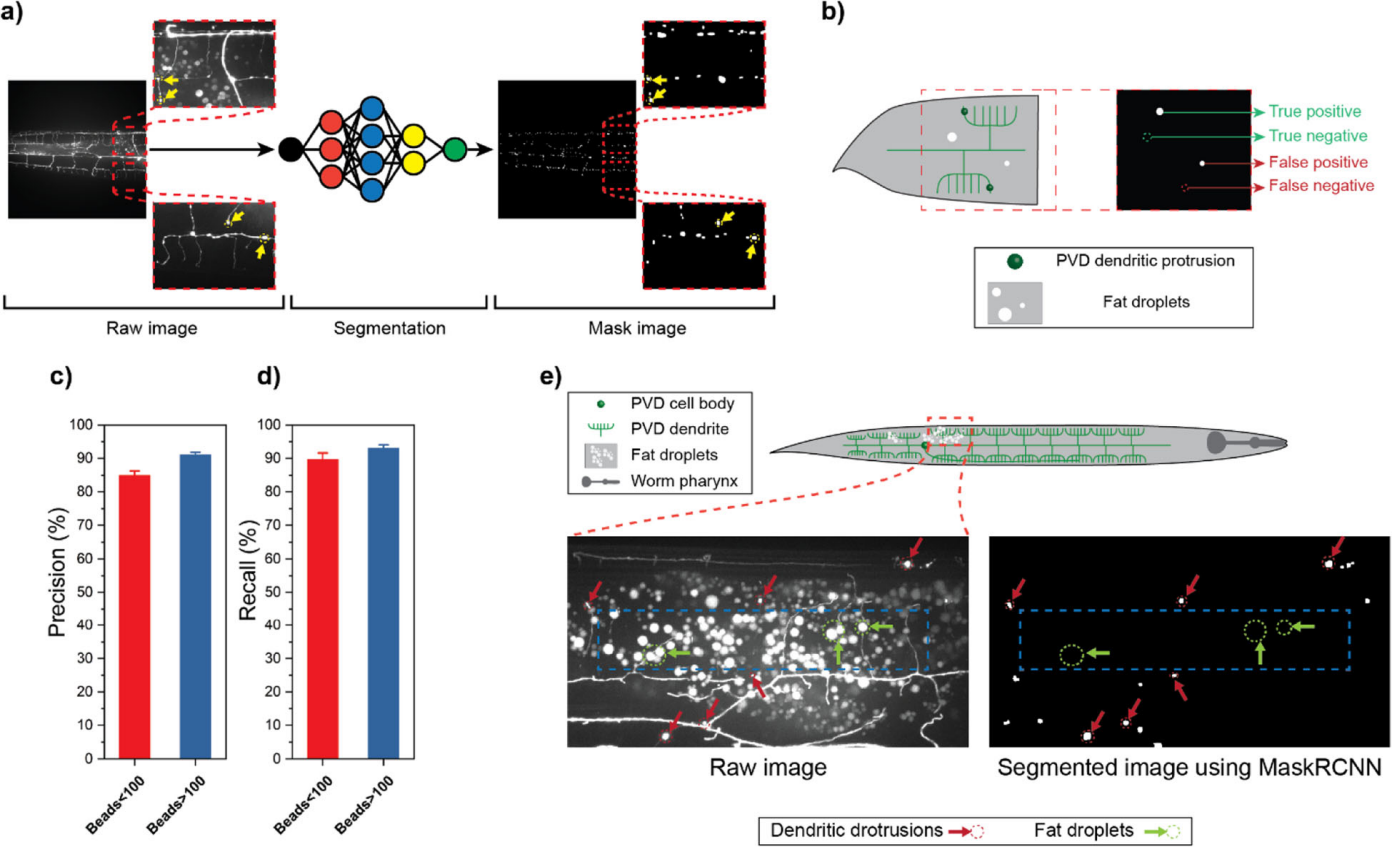
Deep learning approach successfully identifies beads in the PVD neuron. **a** Schematic of segmentation pipeline. Raw 2048 × 2048 images are fed to the trained Mask R-CNN model to perform instance segmentation. Yellow arrows point to neuronal beads. **b** Illustration for true positive, true negative, false positive, and false negative cases used to quantify the performance of instance segmentation. **c**, **d** The performance of the algorithm was examined by defining precision and recall of segmentation where 12 test images were used. Error bars are standard error of mean (SEM). **e** Images showing the algorithm successfully distinguishes bubble-like protrusions (beads) from fat droplets

We adapted the convolutional neural network (CNN) model Mask R-CNN [57] to automatically detect bead protrusions in high-resolution images of nematode dendrites (Fig. 2a). The input to Mask R-CNN is a 1-channel grayscale microscopy image (1024 × 1024 × 1), and the output is a set of predicted bead regions consisting of one binary instance mask (1024 × 1024 × 1) per bead, i.e., a pixel has a value of 1 in the mask when it is part of a bead and 0 otherwise. A tiling procedure was employed to adapt Mask R-CNN for use with 2048 × 2048 × 1 microscopy images (see the “Methods” section), since this image size was sufficient to resolve the smallest bead protrusions. The Mask R-CNN architecture first generates regions of interest (ROIs) using a Faster R-CNN model, composed of a residual network (ResNet-101 [58]) and a feature pyramid network [59]. ROIs are then processed with region proposal and ROI align neural network layers to produce an instance segmentation mask for each detected object. In contrast to thresholding-based methods, which only rely on image intensity for predicting segmentations, CNNs automatically learn and then use hierarchical sets of image features directly from the training data without requiring manual feature engineering. Learning features enable relevant local context to be used in making segmentation predictions, e.g., the shape and size of the bead, what a dendrite looks like, and the proximity of beads to dendrites. We leveraged a transfer learning [60] approach in which Mask R-CNN is pre-trained on a large annotated dataset (ImageNet [61]), and then fine-tuned on a dataset of nematode images that we manually annotated.

The Mask R-CNN algorithm requires a training dataset comprised of raw images of PVD and their corresponding ground truth masks that label the protrusions. The masks were created from raw images using a custom MATLAB code that allows the user to draw around each bead location. A total of 19 images (each with ~ 50–150 beads with an average size of ~ 150 pixels) were manually segmented to compile the training set. In addition, an independent test set was generated with 12 raw images and their associated binary masks. The test set includes diverse images with ~ 30 to ~ 150 beads. These were equally split into images with a low (< 100) and a high (≥ 100) number of beads, to test segmentation consistency. To assess segmentation performance, we quantified precision and recall, described as:

$$ {\displaystyle \begin{array}{l}\mathrm{Precision}=\frac{\mathrm{True}\kern0.17em \mathrm{Positive}}{\mathrm{True}\kern0.17em \mathrm{Positive}+\mathrm{False}\kern0.17em \mathrm{Positive}}\\ {}\mathrm{Recall}=\frac{\mathrm{True}\kern0.17em \mathrm{Positive}}{\mathrm{True}\kern0.17em \mathrm{Positive}+\mathrm{False}\kern0.17em \mathrm{Negative}}\end{array}} $$

In these expressions, true positives are correctly identified beads, false positives are non-bead objects identified as beads, and false negatives are non-identified beads (Fig. 2b). As shown in Fig. 2c, the segmentation precision for the test dataset was 85% and 91% for images with low and high bead numbers, respectively. Similarly, a recall of 90% and 93% was obtained for low and high bead number images, respectively (Fig. 2d). These slight differences could stem from the low number of beads while retaining the same level of objects that can be falsely identified as beads in the first group. The optimized Mask R-CNN algorithm successfully scored 88% in precision and 91% in recall for the entire test set. Thus, this machine learning approach offers consistent unbiased segmentation with high accuracy. To measure the algorithm’s pixel-level accuracy, we calculated the Jaccard index (i.e., intersection over union) of each individual bead in the validation set. The average Jaccard index for all beads was 0.7 (std. dev = 0.15). Furthermore, we show that this index is consistent for animals with drastic beading and with minor beading, as shown in Additional file 1: Fig. S10.

Importantly, precision and recall do not provide information to assess the performance of the model in ignoring objects that can easily be identified as beads (true negatives). In this particular phenotyping problem, this type of objects is prevalent. Autofluorescent lipid droplets can be easily mistaken for neurite protrusions, due to their round shape and location, which can overlap with PVD dendrites in maximum projections. Distinguishing round objects with comparable intensity levels and with similar locations and sizes is a significant challenge. To assess the power of the algorithm to distinguish between the two, we chose 3 images from animals with an abundance of fat droplets that overlapped with dendrites, as part of our training set. As shown in Fig. 2e and Additional file 1: Fig. S1, the algorithm achieved ~ 99% precision in discerning fat droplets from beads, despite their similarities. Prior approaches have addressed this problem by performing dual color microscopy to compare images that show only lipid droplets with images that show the fluorescent reporter [43]. This deep learning approach eliminates the need to perform alternative analyses or dual color microscopy to subtract autofluorescent objects.

### Deep phenotyping of age induced PVD neurodegeneration

**Fig. 3:**
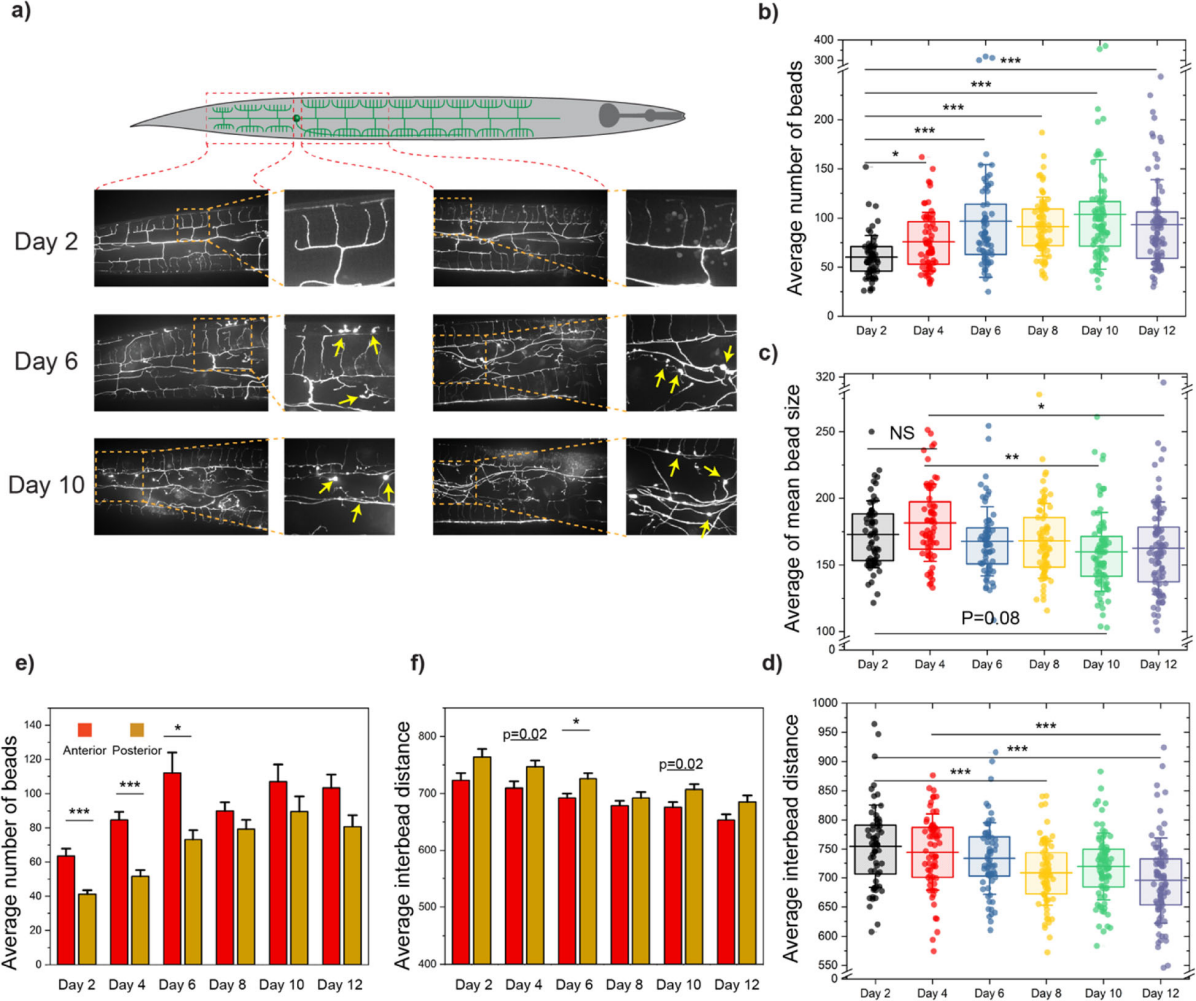
Deep learning allows quantitative analysis of aging-induced morphological changes in PVD. **a** Qualitative inspection of PVD at 3 time points of their life span shows an increase in number of beads throughout the dendrites. Protrusion formation was identified in both anterior and posterior parts of the PVD neuron. Yellow arrows point to neuronal beads. **b**–**d** Average number of beads, average of mean bead size, and average inter-bead distance of both anterior and posterior regions of PVD throughout aging. Lines are the 25th percentile, mean, and 75th percentile. Whisker is the standard deviation. Statistical analysis was performed with one-way ANOVA followed by Tukey’s (**c**) or Steel-Dwass (**b**, **d**) tests for multiple comparison with equal or unequal variance assumptions, respectively, and significance level determined using Bonferroni correction (*α* = 0.016) for multiple feature comparisons. *P < 0.016, **P < 0.001, and ***P < 0.0001. **e**, **f** Average number of beads, and average inter-bead distance of anterior vs. posterior regions of PVD throughout the aging process. Error bar is SEM. Pairwise statistical analysis was performed with *t* test and Bonferroni correction. *P < 0.008 and ***P < 0.0001

The nervous system in *C. elegans* undergoes morphological and functional decline due to aging [14, 18]. Morphological changes in PVD include dendritic outgrowth and beading, which become more common as animals age, as evidenced in Fig. 3a. As previously mentioned, quantitatively investigating beading is difficult as animals can exhibit tens to hundreds of beads with fluorescence intensity levels similar to those of labeled neurons and autofluorescent lipid droplets. Moreover, beading is a highly variable process, and quantification thus requires analysis of large animal populations. We first aimed to quantitatively analyze aging-induced beading in PVD using the deep learning pipeline. Metrics such as average number of beads, size, and inter-bead distance were selected for deeper independent analysis, due to their potential biological significance. These metrics enabled us to examine the morphological changes in PVD. These parameters offered the most descriptive measures which facilitated visualizing the dendritic changes of PVD neuron. Our results (Fig. 3b) show that the average bead count increases from days 2 to 4, 6, and 8 of adulthood. Interestingly, the average number of protrusions does not appear to change significantly afterwards. These results suggest that there may be a saturation point for the beading process, which animals reach at mid-age.

One of the advantages of computer-based image segmentation is that quantification of beading neurodegeneration is not limited to the number of beads. Our post-segmentation MATLAB pipeline enabled extracting additional metrics (a total of 46, Additional file 1) to comprehensively describe the morphological neurodegeneration phenotypes. The average bead size (Fig. 3c) seems to decrease slightly as animals age (days 6–12 vs. days 2–4), which can be explained by an increase in percentage of small beads (area < 100 pixels) (Additional file 1: Fig. S2b). While the size is slightly reduced, the total area occupied by beads increases as nematodes age (Additional file 1: Fig. S2a). These results suggest that the main morphological change induced by aging is an increase in total beading (as measured by number or total bead area), rather than in bead size. The average inter-bead distance (i.e., average of all pairwise distances), which describes how dispersed the beads are, decreases in older populations as expected due to an increase in total number of beads (Fig. 3d). Other metrics that describe bead size and spatial bead distribution (such as 90th percentile of bead size, and percentage of pairwise inter-bead distances < 300 pixels, Additional file 1: Fig. S2d-e) confirmed an overall trend towards accumulation of smaller beads with increased density throughout the neuron in older animals.

To deepen our understanding of aging-induced beading, we compared the patterns exhibited anterior (towards the head) and posterior (towards the tail) to the PVD cell body, since separate images were acquired (Fig. 3a). While both regions exhibit an increase in number of beads (Fig. 3e), this change was more drastic in the anterior section. This difference could be explained either by a higher susceptibility to beading or by the fact that the anterior region occupies larger area, since the posterior is closer to the animal’s tail and is thus more tapered. The average inter-bead distance in the posterior region tends to be larger than in the anterior side (Fig. 3f), as would be expected for a reduced number of beads. As shown in Additional file 1: Fig. S2f, bead morphology appears to be homogeneous, as there is no significant difference in anterior vs. posterior average bead size. Metrics such as the percentage of small beads (< 100 pixels) or the percentage of beads with close neighbors (pairwise inter-bead distances < 300 pixels) did not show any significant differences along the two different sections of PVD (Additional file 1: Fig. S2g-h). This deep learning-based analysis corroborates the neuronal beading reported by Lezi et al., while deepening our understanding of the subtle neurodegenerative patterns that result from aging.

### Acute cold-shock induces morphological changes in PVD neuron

In addition to sensing harsh touch, PVD acts as a thermosensor activated by cold temperatures [62]. Cold-shock has been previously studied as a stressor for *C. elegans* [62–73]. Robinson and Powell identified that animals can survive short (4 h) exposures to acute cold-chock (2 °C), but longer exposures (24 h) result in death for a fraction of the population [74]. Furthermore, Ohta et al. showed that the pre-cold-shock culture temperature is inversely correlated with survival rate (more animals survive cold-shock if previously cultured at lower temperatures) [75]. While the detrimental effects of cold-shock on nematodes’ survival and PVD’s involvement in responding to cold temperatures have been independently studied, the impact of cold-shock exposure on PVD health has not been investigated. To answer this question, we first tested the effects of exposure to cold-shock on PVD morphology, where we identified the appearance of PVD neurite beading. Thus, we sought to examine the effects of acute cold-shock at 4 °C on the structure of PVD through our deep learning phenotyping pipeline.

**Fig. 4:**
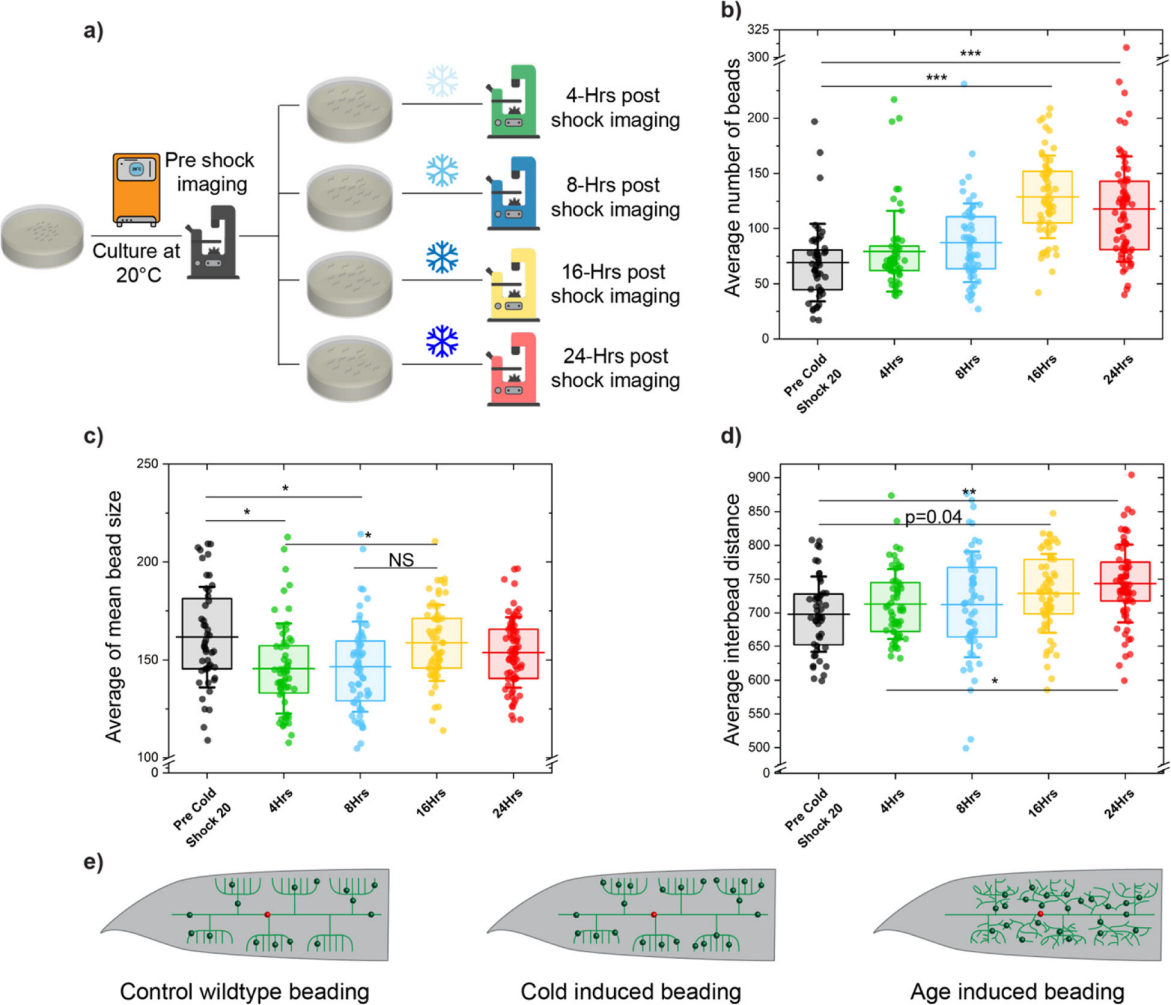
PVD neuronal structure undergoes morphological changes upon exposure to acute cold-shock. **a** Schematic of acute cold-shock assay. Nematodes were cultured at 20 °C until day 2 of adulthood, split into four plates, and cold-shocked for 4, 8, 16, or 24 h. Fluorescence microscopy was conducted before exposure to cold-shock and after specific periods of shock. **b**–**d** Average number of beads, average of mean bead size, and average inter-bead distance of anterior and posterior regions of PVD as nematodes experienced various duration of cold-shock. The lines are the 25th percentile, mean, and 75th percentile. Whisker is the standard deviation. Statistical analysis was performed with one-way ANOVA followed by Tukey’s (**b**, **c**) or Steel-Dwass (**d**) tests for multiple comparison with equal or unequal variance assumptions, respectively, and significance level determined using Bonferroni correction (*α* = 0.016) for multiple feature comparisons. *P < 0.016, **P < 0.001, and ***P < 0.0001. **e** Illustration of distinct beading patterns in aging and acute cold-shock based on the inter-bead distance. Inter-bead distance decreases with aging while it increases with cold-shock. Error bar is SEM

To characterize the relation between cold-shock and beading, we first exposed different *C. elegans* populations to cold-shock for various durations. As shown in Fig. 4a, eggs extracted from gravid hermaphrodites were transferred to NGM plates and cultured at 20 °C until day 2 of adulthood, when pre-cold-shock microscopy was performed. Nematodes were then split into four separate plates and transferred to 4 °C for either 4, 8, 16, or 24 h. Visual inspection of raw images suggested beading increases with longer cold-shock, but is especially evident in populations that were exposed for 16 h or more. Quantitative analysis performed using the trained Mask R-CNN and post-segmentation feature extraction pipeline shows that the number of beads gradually increases with longer periods of cold-shock (Fig. 4b), and is almost doubled after 16 h, as compared to non-exposed animals. Similar to the aging process, beading reaches a saturation point, where no significant change in the number of beads is observed after 16 h. Interestingly, the percentage of small beads (area < 100 pixels) increases after 4 and 8 h of cold-shock, but this effect is not observed after 16 and 24 h (Additional file 1: Fig. S3b). This suggests that new small beads are generated in the first 8 h, resulting in a higher percentage of smaller beads. The drop in percentage of small beads after 16 and 24 h could be due to existing protrusions becoming larger once the number of beads saturate. This fluctuation in percentage of small beads is also reflected in the average size (Fig. 4c), which slightly decreases during the first 8 h of cold-shock and grows after 16 and 24 h. One potential explanation for these observations is that initially new small beads form, but eventually the beading mechanism switches to bead growth rather than bead generation.

Computer-based image processing and quantitative analysis also enabled identifying subtle differences between aging and cold-shock beading patterns. While an increase in bead number was observed in both cases, cold-shock also resulted in an increase in average inter-bead distance (Fig. 4d), in contrast to aging. This counterintuitive result can potentially be explained by the tendency of cold-induced protrusions to form in more distant dendrites (such as 3rd or 4th order branches) of healthy menorahs. With aging, beads are generated evenly throughout the entire neuron, likely as a result of the aging-induced disorganized branching that increases the density of dendrites (where beads are formed) throughout the worm’s body (Fig. 4e). The information extracted from anterior and posterior regions of PVD for nematodes exposed to acute cold-shock shows very similar patterns to aging-induced neurodegeneration (Additional file 1: Fig. S3f-j). Utilizing this deep learning quantitative phenotyping enabled the identification of a previously unknown effect of acute cold-shock on PVD, which is exacerbated with longer exposures. Moreover, this analysis suggests that beading patterns differ for aging and acute cold-shock, suggesting potentially different mechanisms of protrusion formation.

### Post-cold-shock recovery can eliminate PVD dendritic protrusions

**Fig. 5:**
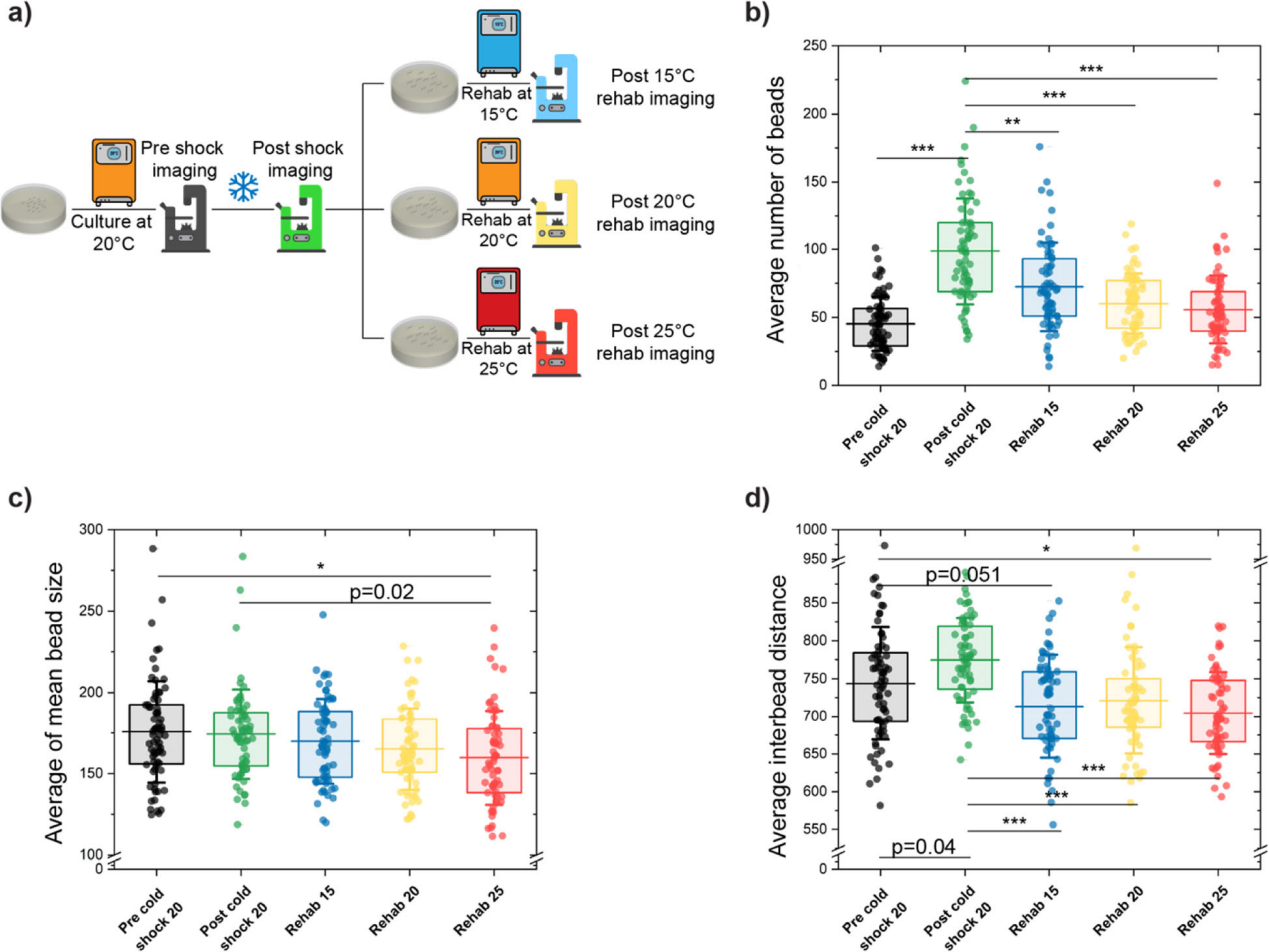
PVD morphological variation phenotypes caused by acute cold-shock are reversible. **a** Schematic of post-cold-shock rehabilitation treatment assay. Nematodes were cultured at 20 °C until day 2 of adulthood and were exposed to cold-shock for 16 h. To perform recovery, the population was split into three plates at either 15 °C, 20 °C, or 25 °C for 1 day. **b**–**d** Average number of beads, average of mean bead size, and average inter-bead distance of PVD neuron as nematodes experienced cold-shock for 16 h and undergo rehabilitation at 3 different temperatures. The lines are the 25th percentile, mean, and 75th percentile. Whisker is the standard deviation. Statistical analysis was performed with one-way ANOVA followed by Tukey’s (**c**, **d**) or Steel-Dwass (**b**) tests for multiple comparison with equal or unequal variance assumptions, respectively, and significance level determined using Bonferroni correction (*α* = 0.016) for multiple feature comparisons. *P < 0.016, **P < 0.001, and ***P < 0.0001. Error bar is SEM

Given the significant increase in number of dendritic protrusions in PVD upon exposure to acute cold-shock, we next sought to determine its potential for regeneration. To test this hypothesis, we designed experiments to characterize PVD beading patterns after acute cold exposure and following a subsequent period under normal culture conditions (referred to as rehabilitation or recovery). As shown in Fig. 5a, we performed 3 “1-day” rehabilitation regimes at 3 different temperatures, selected to cover the entire physiological range (15, 20, and 25 °C). Given that nematodes’ growth rate and life span depend on culture temperature, we expected the population cultured at 25 °C to show a faster recovery rate than those grown at 15 °C. After exposure to 16 h of acute cold-shock, the average number of beads increased by 100% as compared to pre-cold-shock conditions. After 1 day of rehabilitation, we observed a decrease in the number of dendritic protrusions in all three rehabilitation temperatures (Fig. 5b). As expected, populations cultured at 15 °C and 25 °C had the lowest (~ 30%) and highest (~ 50%) recovery, respectively, suggesting that recovery rate is correlated with growth rate.

In addition to a reduction in number, the average bead size slightly decreases after rehabilitation (Fig. 5c and Additional file 1: Fig. S5). This recovery is corroborated by the total area covered by beads (Additional file 1: Fig. S4a), which increases after cold-shock and decreases in all recovery regimes, indicating that bead formation due to cold-shock is reversible. These results suggest that recovery occurs by both bead elimination and a gradual size reduction. To further understand the spatial patterns of cold-shock bead formation, we also explored inter-bead distances. As previously mentioned (Fig. 4c), the average inter-bead distance increased post-cold-shock, suggesting beads are formed in the farthest dendrites. One-day recovery treatment at all three temperatures reduced this metric (Fig. 5d), suggesting that beads on the farthest dendrites are more prone to disappear post-recovery. As expected, the percentage of beads with close neighbors (inter-bead distance < 300 pixels) decreases with cold-shock and increases after recovery (Additional file 1: Fig. S4d). Taken together, these quantitative features suggest that cold-shock induces the formation of beads, particularly in distal regions (as the inter-bead distance increases), and that subsequent culture at physiological temperatures reverts these changes.

In line with previous findings, the anterior region of PVD exhibits a higher number of beads than the posterior region, post-rehabilitation. However, recovery does not appear to favor either side, as both areas show a reduction of beading post-recovery (Additional file 1: Fig. S4f). Likewise, while the posterior region shows higher inter-bead distances than the anterior region, both exhibit a reduction of inter-bead distance post-recovery (Additional file 1: Fig. S4g). The average bead size, percentage of small beads (area < 100 pixels), and percentage of beads with close neighbors (inter-bead distance < 300 pixels) do not show any significant differences between the anterior and posterior regions, either post-cold-shock or post-recovery (Additional file 1: Fig. S4h-j), for most conditions. This suggests that the propensity of the anterior region to increased beading observed with aging is also observed upon cold-shock and after recovery from cold-shock. Taken together, these results indicate that after acute cold exposure, 1 day recovery at different temperatures can almost completely alleviate the induced morphological changes of PVD neuron. In addition, this data suggests that a more efficient recovery can be achieved by rehabilitation at higher temperatures. Finally, it appears that cold-shock preferentially induces beading in the farthest dendrites, but these are also preferentially removed during recovery.

### Pre-cold-shock culture temperature affects the severity of morphological changes

**Fig. 6:**
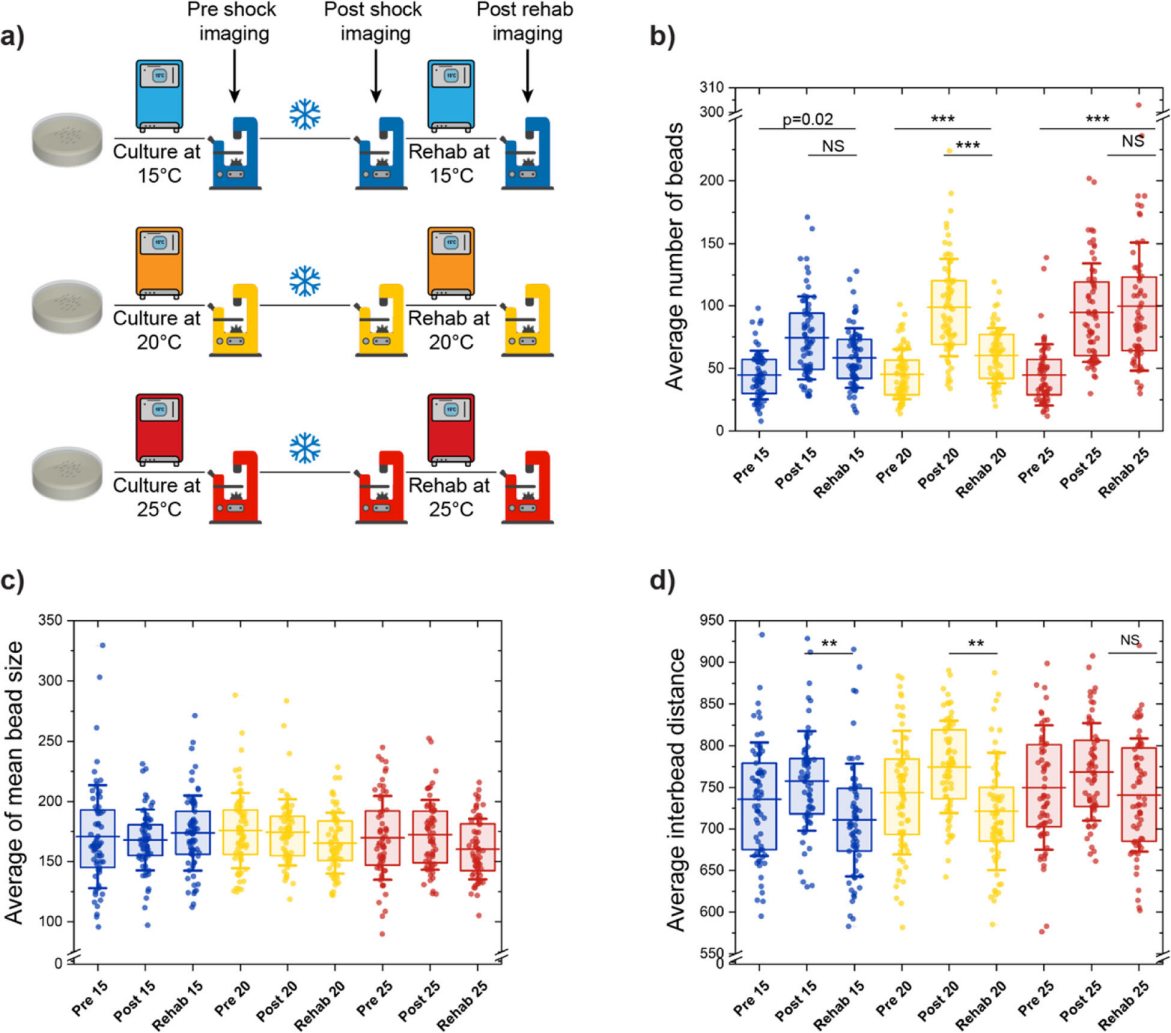
Populations cultured at different temperatures before being exposed to cold-shock show different susceptibility to this stressor. **a** Schematic of the experimental setup to study the effect of pre-cold-shock cultivation temperature. Animals were cultured at 20 °C until young adulthood and then transferred to 15 °C, 20 °C, or 25 °C for 3.5, 2.5, and 1.5 days, respectively. Cold-shock was then performed for 16 h, and rehabilitation was performed for 1 day at the pre-cold-shock temperature. **b**–**d** Average number of beads, average of mean bead size, and average inter-bead distance of PVD for populations that undergo cold-shock as described in **a**. The lines are the 25th percentile, mean, and 75th percentile. Whisker is the standard deviation. Statistical analysis was performed with one-way ANOVA followed by Tukey’s (**d**) or Steel-Dwass (**b**, **c**) tests for multiple comparison with equal or unequal variance assumptions, respectively, and significance level determined using Bonferroni correction (*α* = 0.016) for multiple feature comparisons. *P < 0.016, **P < 0.001, and ***P < 0.0001

Physiological culture temperature is a key environmental factor that affects development, growth, and life span in poikilotherms, such as *C. elegans* [66]. Nematodes habituate to imposed environmental conditions, including temperature [75–79]. Previous studies have identified that after a 4 °C of cold-shock, over 85% of animals cultured at 25 °C die, while most animals cultured at 15 °C survive [75]. These findings motivated us to investigate whether the pre-cold-shock culture temperature plays a role in beading process. To test this, 3 parallel cold-shock/recovery experiments at 3 physiological temperatures were conducted (Fig. 6a) where populations were cultured at 15, 20, and 25 °C for ~ 3.5, 2.5, and 1.5 days, respectively. These animals were then exposed to acute cold-shock at 4 °C and subsequently returned for 1 day to their culture temperature. The difference in culture time prior to cold-shock allowed animals to reach the same developmental stage. Based on prior studies where nematodes cultured at lower temperatures prior to cold-shock have a higher survival rate [75], we hypothesized that lower temperatures would result in less severe morphological changes than high temperatures.

Post-cold-shock behavioral analysis revealed that animals grown at 25 °C were the most affected, as they recovered mobility long after transfer to room temperature (30–40 min), while this time was considerably shorter for animals cultured at 15 and 20 °C. Once animals started crawling, based on qualitative observation, nematodes cultured at 25 °C moved significantly slower than those cultured at lower temperatures. This difference could indicate that worms habituated to a higher temperature may undergo a more drastic shock under cold exposure, although the relevance of locomotion as a metric of shock in the context of PVD is undetermined. These observations suggest that a larger temperature gradient between culture and cold-shock results in increased neuronal damage. As shown in Fig. 6b, the number of beads present after cold-shock and rehabilitation confirms this trend. The average number of beads increases post-cold-shock in all samples, with the smallest change for nematodes grown at 15 °C. The mean bead count after cold-shock reaches the same level for samples cultured at 20 and 25 °C, potentially due to beading reaching a saturation point. This upper limit in number of beads was also observed in neurodegeneration caused by aging and in cold-shock exposure for different periods of time. Interestingly, while populations rehabilitated at 15 and 20 °C show a reduction in number of beads, this effect was not present in those recovered at 25 °C. This could be explained by either a delayed or slower regeneration, or an inability to regenerate for animals cultured at 25 °C. Interestingly, in contrast to animals cultured at 15 and 20 °C, the mean bead size appears to slightly decrease after the rehabilitation regime at 25 °C (Fig. 6c), suggesting that recovery at 25 °C does induce some regenerative effect, although this result was not statistically significant. The regeneration results observed in animals cultured at 20 °C and recovered at 25 °C (presented in the previous section) support the idea that regeneration at 25 °C is possible but is likely slower for the population cultured at 25 °C pre-cold-shock. Such delayed regeneration could stem from the more drastic difference between the baseline and cold-shock temperature. Finally, these experiments corroborate that cold-shock-induced beading occurs in the farthest regions of the neuron, as inter-bead distance increases with cold-shock, and is then reduced after rehabilitation for all culture temperatures (Fig. 6d). The percentage of small beads (area < 100 pixels) and the percentage of beads with close neighbors (inter-bead distances < 300 pixels) (Additional file 1: Fig. S6b,d) also show a reversal of the cold-shock exposure effect in all three physiological temperatures.

Consistent with our previous results, the anterior region of PVD showed a higher number of protrusions than the posterior (Additional file 1: Fig. S6f). Both regions recapitulate the trends observed for pre-cold-shock, post-cold-shock, and post-rehabilitation in the entire animal. The anterior region consistently exhibits ~ 20–100% higher number of beads than the posterior, with pre-cold-shocks showing the largest difference. The average bead size does not show differences between these regions (Additional file 1: Fig. S6h). However, similar to previous experiments, the protrusions are more densely distributed in the anterior part, as is expected for a higher number of beads (Additional file 1: Fig. S6g). The results from this assay support our hypothesis that the culture temperature impacts how nematodes respond to acute cold-shock. Animals cultured at 15 °C exhibited the least morphological changes and faster recovery, while those grown 25 °C showed more drastic beading and slower rehabilitation rate. This difference in response indicates that the magnitude of the cold-shock (based on the baseline temperature) correlates with the induced morphological alteration through a yet unknown mechanism.

### Predicting biological status using deep quantitative classification

**Fig. 7:**
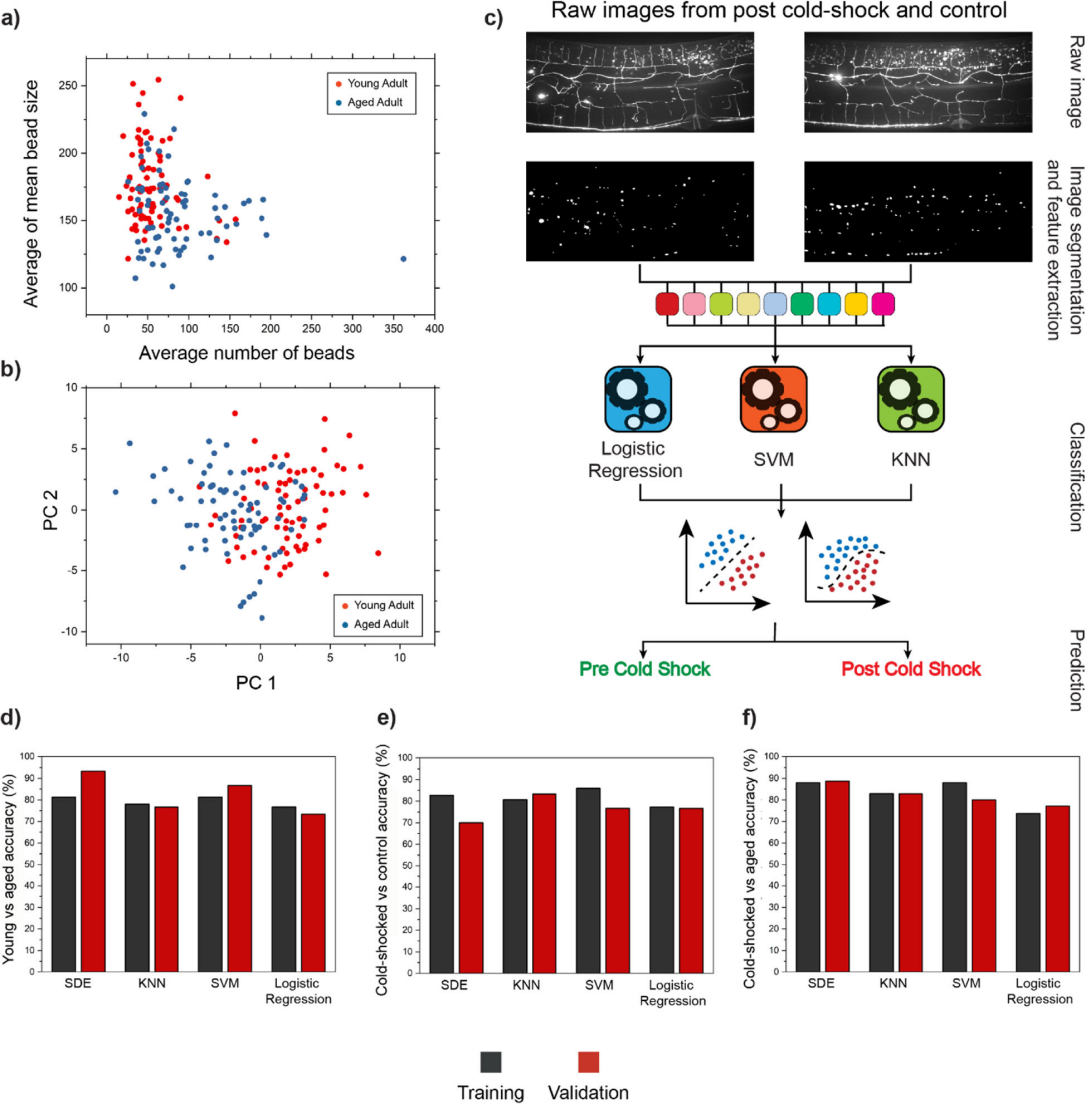
Biological status of a nematode can be predicted based on PVD neuron’s health. **a** Average of mean bead size vs. the average number of beads for young and aged nematodes. Age-induced PVD degeneration patterns are complex, and two metrics are not sufficient to accurately classify the two populations. **b** Principal component analysis (PCA) for young and aged adults does not enable distinguishing young and aged groups, based on the two first principal components. **c** Schematic of the pipeline for computer-based machine learning models to predict the nematode’s biological status based on the morphological structure of PVD. Raw images are fed to Mask R-CNN algorithm to obtain binary mask, which is then used to extract the 46 metrics. Multiple models were trained based on these 46 metrics and tested on separate datasets. **d**–**f** Classification accuracy for young vs. aged, cold-shocked vs. control, and cold-shocked vs. aged nematodes. *N*_T_ young = 50, *N*_T_ old = 100, *N*_V_ young = 10, and *N*_V_ old = 20 for young vs. aged classification. *N*_T_ cold-shocked = 75, *N*_T_ control = 75, *N*_V_ cold-shocked = 15, and *N*_V_ control = 15 for cold-shock vs. control classification. *N*_T_ cold-shocked = 75, *N*_T_ aged = 100, *N*_V_ cold-shocked = 15, and *N*_V_ aged = 20 for cold-shocked vs. aged classification. (*N*_T_, numbers for training set; *N*_V_, numbers for validation set). SDE, subspace discriminant ensemble; KNN, *K*-nearest neighbor; SVM, support vector machine

The quantitative analysis of beading induced by aging and cold-shock indicates that the patterns of PVD morphological changes are different. To further investigate the morphological changes observed, we took advantage of the rich information obtained from the Mask R-CNN segmentation and feature extraction pipeline, which includes all 46 metrics. Through visual inspection of the raw images, as well as the quantitative analysis of the beading patterns, it is clear that beading phenotypes cannot be fully described with a single feature, such as number of beads. Furthermore, there is significant variability within a population. As shown in Fig. 7a, a large fraction of aged animals exhibit less than 70 beads, which is considerably lower than the average of the population and is closer to the number of beads for young individuals. Likewise, some young animals showed more than 70 beads, which is significantly higher than the average of the population. The same variability was observed in cold-shock experiments, suggesting that the number of beads does not offer a comprehensive description about biological status of a nematode. Combining two metrics such as number of beads and average bead size still does not provide enough information to distinguish between young and aged adults (Fig. 7a).

Given that beading patterns relay information about the health state of PVD, we reasoned that beading phenotypes could be used to predict the biological state of the animals. To test this hypothesis, we sought to incorporate all 46 metrics extracted from each image in a classification model. In a first attempt, as shown in Fig. 7b, we performed PCA (principal component analysis) on the 46 metrics. Two principal components (PC1 and PC2) explain 46% of the total variance and are unable to accurately differentiate nematodes from these two stages in their life span. Thus, we aimed to test the ability of classification models to distinguish young and old nematodes using the metrics extracted from PVD beading patterns. As shown in Fig. 7c, animals from different groups (e.g., pre- and post-cold-shock) can exhibit very similar beading patterns. Successful predictive models would prove the presence of subtle patterns that can only be described using multiple metrics. We first developed a classification model to distinguish young vs. old adults. To create a labeled training set, data from the posterior side of PVD for worms younger than 4 days old were grouped together while the second class was comprised of information from nematodes older than 4 days old. An independent validation dataset was then generated to test classification accuracy. It should be noted that these two classes are more difficult to distinguish than comparing day 2 vs. day 12 animals (i.e., the youngest vs. the oldest samples). We tested four classification algorithms: subspace discriminant ensemble (SDE), support vector machines (SVMs), logistic regression, and *K*-nearest neighbors (KNNs). Two models, SDE and SVM, achieved both training and validation accuracies above 80%, with the validation accuracy of SDE reaching 90% (Fig. 7d). For age-based classification, the information acquired from the PVD anterior side was also used to train separate models leading to training and validation accuracies higher than 80% (Additional file 1: Fig. S7a). In addition, the area under curve (AUC) of the receiver operating characteristic (ROC) curve for both anterior and posterior section reached 0.89 and 0.88, respectively (Additional file 1: Fig. S8). These results suggest that age-induced PVD neurodegeneration causes subtle morphological changes that can only be captured using quantitative deep phenotyping. We also sought to analyze the capability of our trained classifiers in correctly classifying aged nematodes with low number of beads. As shown in Additional file 1: Fig. S9, the trained SDE classifier is capable of identifying aged nematodes even when these appear young, based on their low number of beads, suggesting that other metrics are relevant to differentiate distinguish these two groups. To gain some insight regarding the relevant metrics to distinguish these populations, a stepwise logistic regression was performed and 5 metrics were found to be important. These include the following: percentage of beads with area smaller than 100 pixels (metric 16), standard error of mean for inter-bead distance (metric 20), standard deviation of mean bead intensity (metric 35), 90th percentile of bead intensity (metric 37), and 25th percentile of bead intensity. As shown in Additional file 1: Fig. S9, although the data points for young and aged nematodes overlap for metrics 16, 20, and 37, the classifier is capable of achieving 90% accuracy in distinguishing them, suggesting the beading phenotype requires integration of information from multiple metrics. Similarly, we tested models for classifying nematodes exposed to cold-shock from those that did not experience this stressor. The training and validation set for this analysis was comprised of data from cold-shock performed at all three pre-cold-shock temperatures, and as shown in Fig. 7e, ~ 80% classification accuracy was obtained both in training and validation. Since differences between degenerated (i.e., old or cold-shocked) and healthy (young or non-cold-shocked) animals have been shown, it was expected that these populations are distinguishable. However, given the significant variability in each population, the high classification accuracy obtained was surprising and points to consistent phenotypic patterns exhibited upon morphological alteration that are not evident to visual inspection.

To further test the power of our deep phenotyping pipeline, we next investigated potential differences in PVD exhibited upon aging and acute cold-shock. We compiled data from the anterior and posterior part of the PVD from aging and cold-shock assays to generate training and validation sets. As shown in Fig. 7f, the SDE model reaches ~ 90% training and validation accuracy for the anterior and ~ 80% for the posterior regions (Additional file 1: Fig. S7c). This difference in classification accuracy could stem from the anterior part of PVD undergoing stronger beading patterns than the posterior. Notably, these results indicate that these two stressors cause distinct morphological patterns which can be captured by in-depth quantitative analysis. To further elucidate the differences between these two stressors, we sought to identify the metrics used to distinguish these two groups. The training dataset was used to fit a stepwise logistic regression model to identify the metrics most important in the classification process. Interestingly, the most important metrics were average inter-bead distance (metric 17) and percentage of beads with inter-bead distance below 150 pixels (metric 21). These findings are compatible with the observed trends in average inter-bead distance between aging and acute cold-shock. In addition to these two metrics, median bead size (metric 25), max bead size (metric 26), and median bead intensity (metric 32) were among the metrics incorporated by the stepwise logistic regression model. As a last test, we sought to establish whether the differences in bead patterning between the anterior and posterior part of the PVD could be used to classify images of each class. An accuracy of ~ 85–90% was achieved from different models, confirming underlying beading pattern differences between these two regions of the neuron (Additional file 1: Fig. S7d). The developed classification models are a powerful tool to identify potential differences in beading patterns caused by various environmental stressors (cold-shock or aging).

## Conclusions

Neurons undergo degeneration during the aging process. In *C. elegans*, PVD, a neuron responsible for mechanosensation and thermosensation, experiences morphological and functional changes as animals age [37, 39]. Prior studies have identified changes in dendrite morphology characterized by disorganization in the menorah-like dendritic arbors [33, 34]. In addition, Lezi et al. identified that aging results in the formation of protrusions (or beads) along PVD dendrites [39]. However, analyzing such morphological changes is challenging. Manual inspection to quantify the number of protrusions is time-consuming, labor-intensive, low-throughput, and subject to human bias. In addition, manual counting provides limited information and makes thorough analysis of the complex phenotypes acquired in fluorescence images unfeasible. In order to track morphological changes that PVD undergoes with degeneration, we integrated a cutting-edge deep learning technique to segment the protrusions that form along PVD. This technology decreased the time required to process each image from 3 h to less than a minute, while eliminating the human bias in analyzing the data. In addition, a secondary algorithm was developed to extract 46 different metrics that make up a comprehensive phenotypic profile that describes the beading patterns.

We implement a convolutional neural network-based algorithm (Mask R-CNN) to carry out challenging image segmentation, unfeasible with traditional image processing approaches. The algorithm segmentation precision and recall achieved 88% and 91%, respectively. An important advantage offered by this technology (which cannot be quantified using the metrics above) is its capability to distinguish autofluorescent lipid droplets from actual protrusions, in spite of their remarkable similarities in shape, intensity, and location. High recall and precision achieved using Mask R-CNN can be further improved in future work by implementing enhanced architects, such as Mask Scoring R-CNN [80]. The in-depth quantification of PVD morphology enabled by this technology revealed subtle neurodegenerative changes induced by aging and upon exposure to acute cold-shock. With this approach, we identified an increase in the number of beads formed along PVD as animals aged, recapitulating earlier work by Lezi et al. [39]. In addition, the reduction in average bead size and inter-bead distance quantified in later points of the nematode’s life span suggested that the protrusions formed due to aging tend to be small and appear close to each other.

Prior work has focused on the effect of acute cold-shock on a population’s survival and on PVD degeneration independently [37, 39, 63, 75]. However, the impacts of acute cold-shock on PVD were still unexplored. We sought to test the effect of acute cold-shock on PVD by exposing populations of worms to 4 °C and subsequently quantifying the protrusions generated as a result. We demonstrate that exposure to cold-shock for 16 h or more induces bead formation in PVD. In contrast to the beading patterns induced by aging, the average inter-bead distance increased in animals as a result of cold-shock, a counterintuitive result as an increased bead density is expected with a higher number of beads. This finding, however, can be explained by the formation of beads in the farthest regions of the neuron. These results were the initial signs of aging and cold-shock inducing phenotypically distinct beading patterns. We next sought to study the regenerative potential of PVD post-cold-shock. Thus, populations of worms exposed to cold-shock were transferred to 3 different temperatures (15, 20, and 25 °C) for a day of recovery. Interestingly, a decrease in the number of beads was observed after the rehabilitation in all 3 temperatures, while the population cultured at 25 °C exhibited the greatest decrease. The increased inter-bead distance induced by cold-shock was reversed in all three temperatures. These results suggest that bead formation due to cold-shock is a reversible process, at least at the earlier stages of adulthood. We also investigated whether culture temperature impacts the severity of bead formation due to cold-shock. Our data suggested that populations cultured at lower temperatures experience less drastic morphological changes, while those cultured at higher temperatures undergo more severe damage.

Finally, we use our deep phenotyping approach to predict the biological status of nematodes based on 46 metrics extracted from the images. We tested multiple algorithms (SVM, KNN, SDE, and logistic regression) to classify young and old adults, cold-shocked and non-shocked nematodes, and cold-shocked and aged worms. These models achieved ~ 85% classification accuracy, indicating distinct beading patterns result from different stressors. Importantly, this classification method, which relies on multiple descriptive metrics of beading patterns, enables deeper exploration of the relevant parameters that describe the biological status of the neuron and its particular beading pattern. These promising results suggest that this approach can be used in future studies to characterize beading patterns associated with other conditions or environmental stressors. While the nature of the beads is still unclear, this approach will be crucial in understanding their role, composition, and generation mechanisms, by applying it in genetic or drug screens, and to test the beading patterns formed under other conditions.

In this work, we developed a computer-based comprehensive pipeline to study the dynamics of PVD morphological changes in a high-content, automated manner. Our quantitative analysis enabled interrogating the morphological changes that PVD undergoes under different scenarios, leading to deeper understanding of neuronal degeneration. Through this deep phenotyping pipeline, we identify a new environmental stressor (cold-shock) that induces morphological changes characterized by beading and reveal distinct neurodegeneration patterns induced by aging. The presented results are evidence that this high-content phenotyping technology can be used to characterize subtle and noisy beading patterns with differences among stressors unnoticeable to the human eye. This pipeline is a promising approach to further explore the mechanisms underlying of beading in these and other contexts (such as oxidative stress, dietary restriction, and neurodegenerative disease models), to understand the differences that lead to distinct aging and cold-shock-induced morphological changes, and to identify whether beads are a result of loss of neuronal integrity or could act as a protective mechanism. In addition, with minor modifications, this pipeline could be implemented to investigate beading and protrusion forming along other neurons such as ALM and PLM. The application of these quantitative methods can be further broadened by investigating beading and blebbing as a result of degeneration [23].

## Methods

### Worm culture

The *C. elegans* strain used in this work is NC1686 (wdls51 [F4H12.4::GFP + unc-119(+)]), which expresses GFP in PVD. All populations were cultured on solid nematode growth media (NGM) plates. For aging experiments, 12 mg of fluorodeoxyuridine (FUdR) was added to 1 L of media (50 μM). Animals exposed to this concentration of FUdR produced non-viable eggs. For cold-shock experiments, plates without FUdR were used since experiments took place in 4 days. Age-synchronized populations were obtained by extracting eggs from gravid hermaphrodites using a bleaching solution (1% NaOCl and 0.1 M NaOH). Eggs were then transferred to NGM plates seeded with *Escherichia coli* OP50. M9 buffer (3 g KH_2_PO_4_, 6 g Na_2_HPO_4_, 5 g NaCl, and 1 mL of 1 M MgSO_4_ in 1 L of water) with 5 μM Triton X-100 was used to transfer worms.

### Microscopy

Animals were mounted on 2% agarose pads on glass slide. Agarose pads were placed at room temperature overnight before microscopy. A drop of 10 mM tetramisole in M9 buffer was added for immobilization. Images were acquired on a Leica DMi8 equipped with a spinning disk confocal head (CrestOptics X-light V2) and a Hamamatsu Orca-Fusion camera using a × 63 objective. The illumination source is a Laser Diode Illuminator (89 North LDI). The imaging settings were maintained constant for all images (exposure time of 60 ms and laser power at 50%). Due to small field of view provided by the high-magnification × 63 objective (NA = 1.40), two sections of each worm (anterior and posterior of PVD cell body) were imaged separately to cover larger area of the body. Images were acquired as z-stacks of 31 slices taken 1 μm apart. The final raw images used in this study were maximum projections of the z-stacks taken at every 1-μm step.

### Image segmentation and analysis

The inputs to the Mask R-CNN machine learning algorithm trained for this study were 2048 × 2048 maximum projection PNG images. Images were preprocessed before being fed to the algorithm using MATLAB image processing toolbox (imadjust function) to equalize the image contrast throughout the dataset. We modified the Mask R-CNN implementation open-sourced by Matterport Inc. under the MIT license [81] using Python3, Keras [82], and Tensorflow [83]. During training, each 2048 × 2048 × 1 image and its set of corresponding binary instance masks were split into 9 overlapping tiles of size 1024 × 1024 × 1. Symmetric padding was used on the boundary of each 2048 × 2048 × 1 image to ensure 9 tiles of size 1024 × 1024 × 1 were obtained. A total of 19 images of size 2048 × 2048 × 1 were contained in the training data, and 12 images were contained in the testing data. The 19 images of size 2048 × 2048 × 1 were each tiled into 9 tiles of size 1024 × 1024 × 1, resulting in a total of 171 training images of size 1024 × 1024 × 1 with its corresponding instance masks. The 19 training images contained a total of 1642 beads with corresponding instance segmentation masks, ranging from 6 beads to 293 beads per image with a median of 65 beads per image. The 12 images in the testing set contained a total of 965 beads with corresponding instance segmentation masks, ranging from 23 to 157 beads per image with a median of 77 beads per image. The Mask R-CNN head was trained for 20 epochs, and the entire model was trained for 400 epochs, starting from pre-trained ImageNet weights. The training data were augmented inline during training using random combinations of left-right and up-down flips, 90° rotations, and affine shearing. From the 171 training images, approximately 20% (34 images) were held out as a validation set used to compute a validation loss. The model with the lowest validation loss after 400 epochs was used for predicting instance masks. Importantly, our ability to train a deep neural network for instance segmentation on only 171 training images relied on leveraging a combination of transfer learning with pre-trained weights and data augmentation. The strong use of data augmentation was observed to enable robust training on only a few training samples in previous deep learning models, e.g., the seminal work on the U-net architecture [50]. We note that we also experimented with Gaussian blur and contrast augmentations, but found that random rotations and shearing were sufficient for our training dataset, likely because the combination of microscopy parameters and the use of the imadjust function for contrast adjustment ensured uniform image sharpness and brightness across all experiments. The use of inline augmentations over 420 epochs resulted in an effective training set size of ~ 57k images containing a total of ~ 552k beads with corresponding instance masks, which were used to train the Mask R-CNN model parameters (~ 47.8 million). The trained Mask R-CNN model was used to predict instance masks by similarly tiling the testing images. Predictions were made sequentially on 9 tiles from the top left to the bottom right of each image, and newly predicted instance masks were kept only if they did not overlap with any previously predicted mask by more than 30%. Only objects yielding a predicted probability greater than 0.7 of being in the foreground or “bead” class were kept. The threshold of 0.7 was chosen based on optimization of the precision metric (see the “Training the Mask R-CNN algorithm to perform complex image segmentation” section) on the training dataset. The binary masks acquired by performing image segmentation using the Mask R-CNN were then coupled with raw images and fed to secondary MATLAB-based algorithm to extract metrics describing the morphology of neuronal beads.

MATLAB algorithms for feature extraction is publicly available on github: github.com/asanmiguel/Beading

### Aging assay

Eggs extracted from gravid hermaphrodites were transferred to a seeded plate and maintained at 20 °C until the population reached late L4 stage and then transferred to an FUdR plate. FUdR plates were checked daily to ensure no viable eggs or progeny were produced. During the first 7–8 days of adulthood, nematodes were transferred to a fresh FUdR plate on a daily basis to provide worms with sufficient food specially during their early adulthood. Every 2 days, a subset of nematodes was picked to perform high-resolution microscopy.

### Cold-shock assay

The cold-shock experiments were designed to be conducted in ~ 4 days, which included pre/post-cold-shock microscopy and rehabilitation. For the first two cold-shock assays, eggs extracted from gravid hermaphrodites were transferred to NGM plates and cultured for 4–5 days at 20 °C until they reach day 2 of adulthood, when pre-cold-shock microscopy was performed. Cold-shock was performed by transferring plates to a 4 °C refrigerator for the designated amount of time. Plates were then placed at room temperature for 1 h before performing post-cold-shock microscopy. This hour-long rehabilitation allowed nematodes to regain their mobility. For the tests where rehabilitation was needed, plates were transferred to designated temperature (15 °C, 20 °C, and 25 °C) for 1 day before post-rehabilitation microscopy was performed. For the pre-cold-shock culture temperature effect assay, nematodes were cultured at 20 °C until reaching young adulthood. Subsequently, the three populations were transferred to 15 °C, 20 °C, and 25 °C incubator and cultured for 3.5, 2.5, and 1.5 days before performing pre-cold-shock microscopy on each population (to ensure all three samples reach the same developmental stage). Populations experienced 16 h of cold-shock at 4 °C prior to post-cold-shock microscopy. The samples were then transferred back to the temperature they were cultured at before cold-shock for 1 day to examine the post-shock recovery.

### Principal component analysis and classification

Principal component analysis (PCA) based on correlation was performed using JMP Pro 14 software. For this analysis, a dataset comprised of 150 images (half from animals younger than 4 days and half from animals older than 4 days old) was generated. All 46 metrics extracted from images were incorporated in the analysis. The first two principal components explained 46% of the variance. Classification of biological status was conducted using MATLAB Classification Learner App. For all training sessions, all 46 metrics extracted from images were incorporated to train the models, and used 5-fold cross-validation was carried out. A separate validation set was used to test performance.

## Supplementary information

**Additional file 1.**

## Data Availability

The datasets used and/or analyzed during the current study are available from the corresponding author on reasonable request.

## Abbreviations

CNN: Convolutional neural network
KNN: *K*-nearest neighbor
PCA: Principal component analysis
SDE: Subspace discriminant ensemble
NGM: Nematode growth media
ROI: Region of interest
SVM: Support vector machine
AUC: Area under curve
ROC: Receiver operating characteristic
