# Supplementary material for: Deep learning-enabled analysis reveals distinct neuronal phenotypes induced by aging and cold-shock
Source: BMC Biol. 2020 Sep 23;18:130. doi: 10.1186/s12915-020-00861-w (PMC7510121; doi:10.1186/s12915-020-00861-w)
Supplement: Supplementary file 1 — Additional file 1. [file 12915_2020_861_MOESM1_ESM.docx]

**Additional File 1: Deep learning-enabled analysis reveals distinct neuronal phenotypes induced by aging and cold-shock**

**Sahand Saberi-Bosari^1^, Kevin Flores^2^, Adriana San-Miguel^1^**

^1^ Department of Chemical and Biomolecular Engineering, North Carolina State University, Raleigh, North Carolina 27606, USA

^2^ Department of Mathematics, North Carolina State University, Raleigh, North Carolina 27606, USA

**46 metrics extracted from each image:**

1. Number of beads
2. Total area occupied by beads
3. Average bead size
4. Standard deviation of bead size
5. Standard deviation of mean of bead size (Standard deviation/Mean)
6. Standard error of mean for bead size
7. 90^th^ percentile of bead size
8. 75^th^ percentile of bead size
9. 50^th^ percentile of bead size
10. 25^th^ percentile of bead size
11. Average bead size for beads larger than 100 pixels
12. Average bead size for beads smaller than 100 pixels
13. Number of beads with area larger than 100 pixels
14. Number of beads with area smaller than 100 pixels
15. Percentage of beads with area larger than 100 pixels
16. Percentage of beads with area smaller than 100 pixels
17. Average inter-bead distance
18. Standard deviation of inter-bead distance
19. Standard deviation of mean inter-bead distance (Standard deviation/Mean)
20. Standard error of mean for inter-bead distance
21. Percentage of beads with inter-bead distance less than 150 pixels
22. Percentage of beads with inter-bead distance less than 300 and greater than 150 pixels
23. Percentage of beads with inter-bead distance less than 450 and greater than 300 pixels
24. Percentage of beads with inter-bead distance greater than 450 pixels
25. Median of bead size
26. Maximum of bead size
27. Average size for beads larger than 100 pixels/ Average size for beads smaller than 100 pixels
28. Mean size for smallest beads (Smallest beads are smaller half of the beads)
29. Mean size for largest beads (Largest beads are larger half of the beads)
30. Mean size of largest beads/ Mean size of smallest beads
31. Average of mean bead intensity
32. Median of mean bead intensity
33. Max of mean bead intensity
34. Standard deviation of mean bead intensity
35. Standard deviation of mean bead intensity (Standard deviation/Mean)
36. Standard error of mean of bead intensity
37. 90^th^ percentile of mean bead intensity
38. 75^th^ percentile of mean bead intensity
39. 50^th^ percentile of mean bead intensity
40. 25^th^ percentile of mean bead intensity
41. Mean intensity of smallest beads
42. Mean intensity of largest beads
43. 90^th^ percentile of inter-bead distance
44. 75^th^ percentile of inter-bead distance
45. 50^th^ percentile of inter-bead distance
46. 25^th^ percentile of inter-bead distance


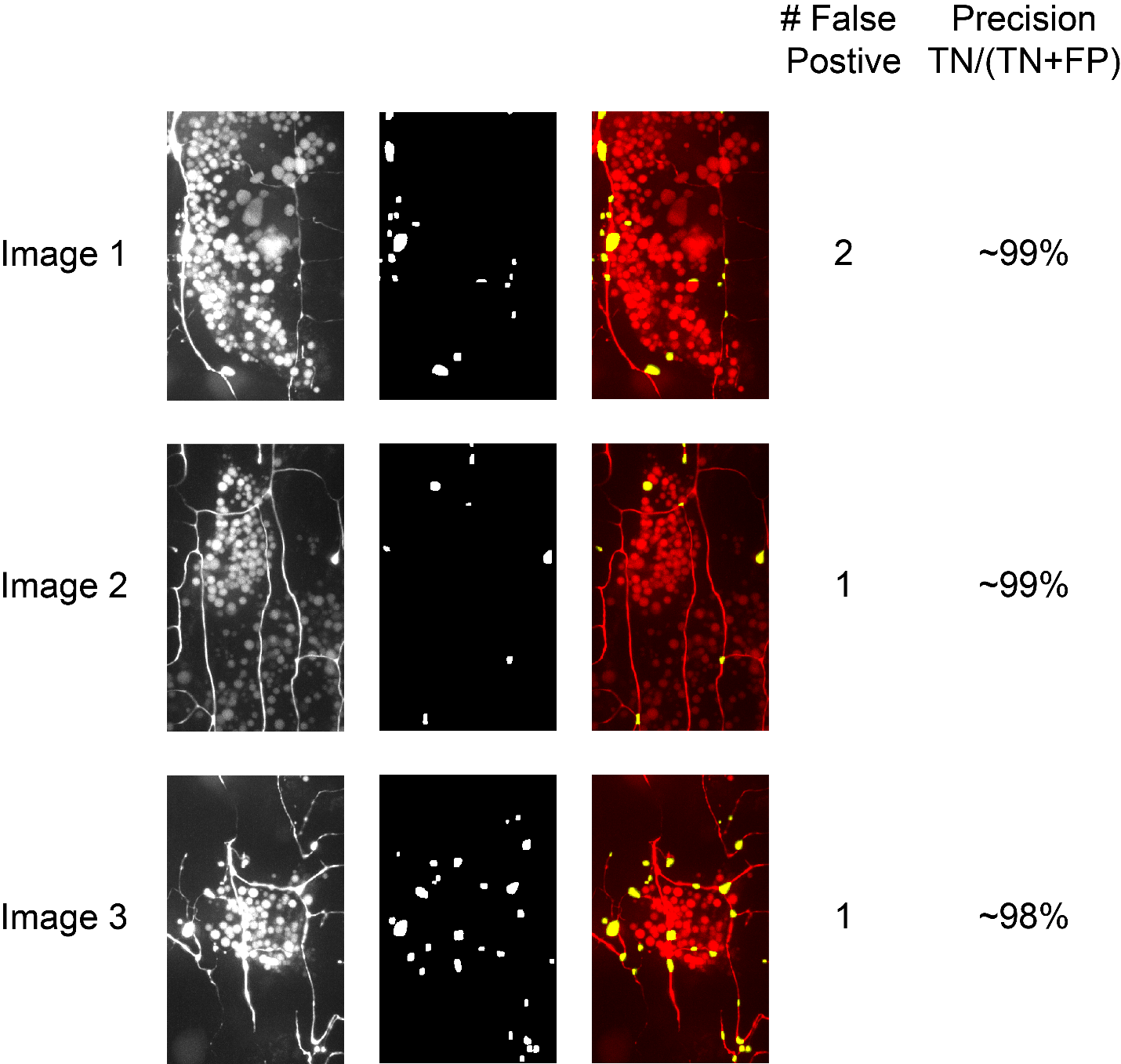


**Additional File 1: Figure S1.** Quantification of the competency of the trained MaskRCNN algorithm in segmentation and distinguishing.


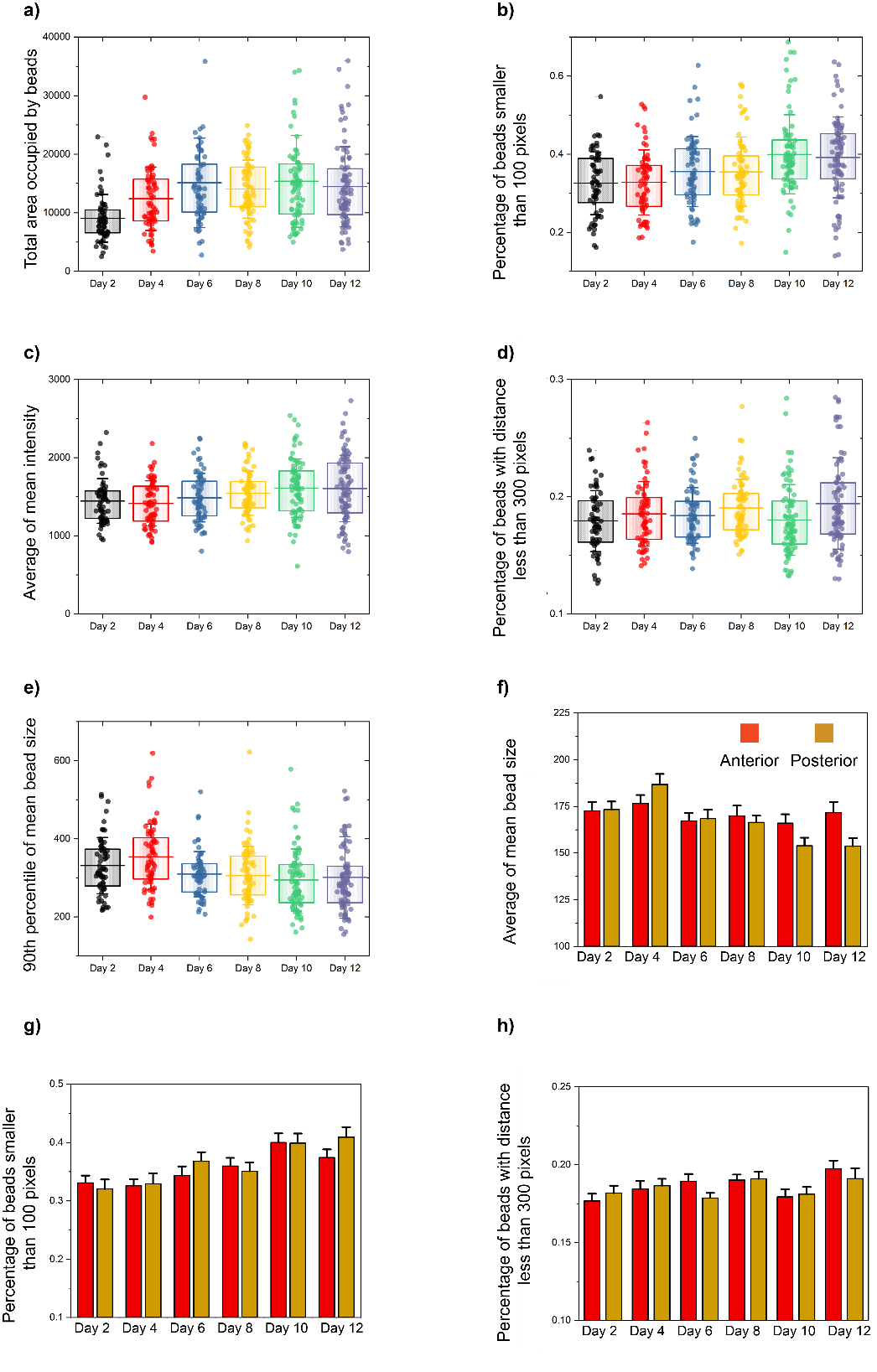


***Additional File 1: Figure S2.*** *Age-induced degeneration causes morphological variation on PVD structure.* ***a)*** *Total area of PVD neuron covered with beads.* ***b)*** *Percentage of beads with size lower than 100 pixels.* ***c)*** *Average of the mean intensity of the beads.* ***d)*** *The percentage of inter-bead distances lower than 300 pixels.* ***e)*** *90^th^ percentile of beads fluorescence intensity. Lines are 25^th^ percentile, mean, and 75^th^ percentile. Whisker is the standard deviation.* ***f-h)*** *Average of mean bead size, percentage of beads with inter-bead distance less than 300 pixels, and percentage of beads with size lower than 100 pixels for anterior versus posterior part of the PVD neuron.*

*
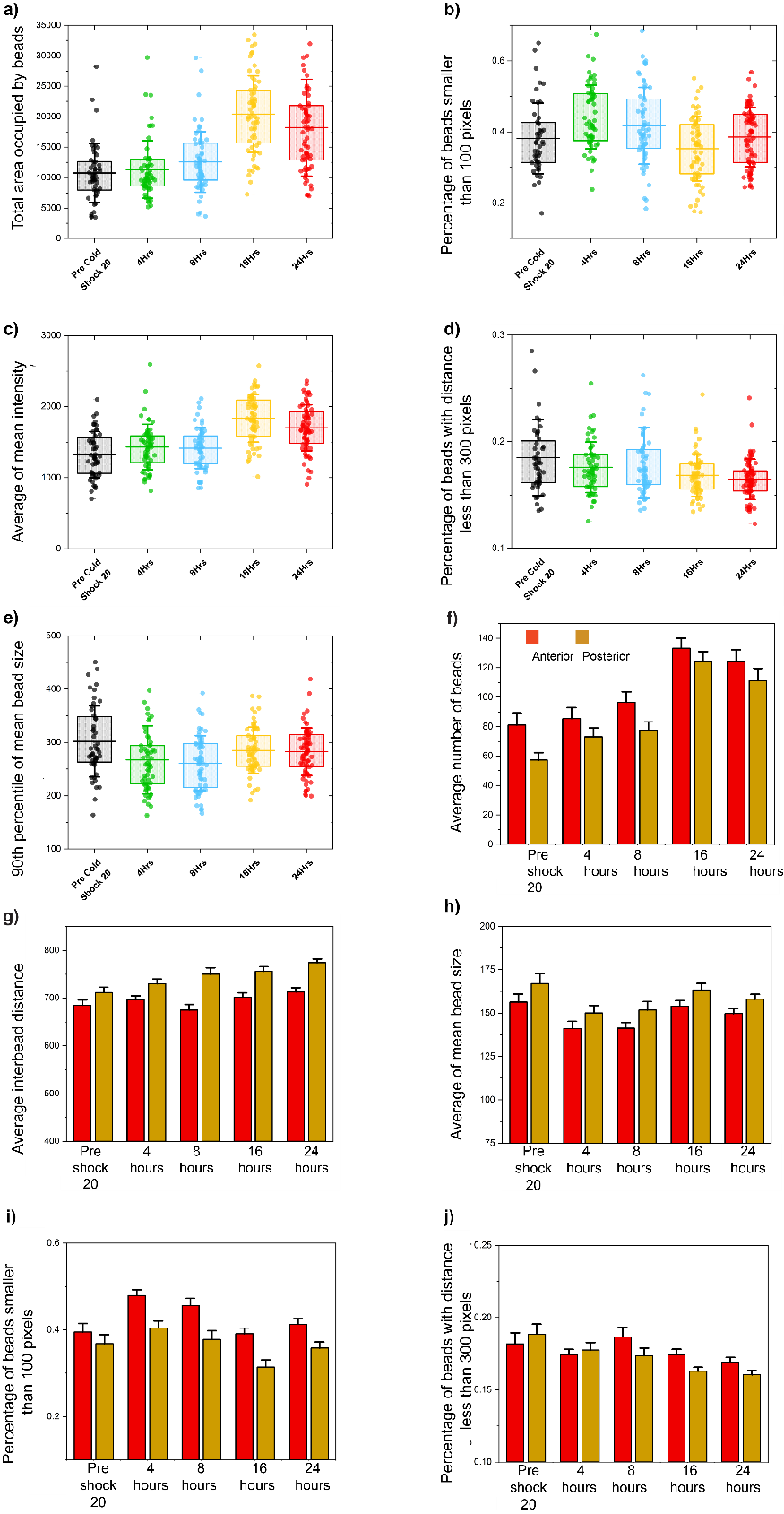
*

**Additional File 1: Figure S3.** PVD neuronal structure undergoes morphological changes upon exposure to acute cold-shock. **a)** Total area of PVD neuron covered with beads. **b)** Percentage of beads with size lower than 100 pixels. **c)** Average of the mean intensity of the beads. This line is a transcriptional line; thus, the change is the representative of the fluctuation in concentration on promoter along beads. **d)** The percentage of inter-bead distances lower than 300 pixels. **e)** 90^th^ percentile of beads fluorescence intensity. The lines are 25^th^ percentile, mean, and 75^th^ percentile. Whisker is standard deviation. **f-j)** Average number of beads, average inter-bead distance of anterior versus posterior parts of PVD upon cold-shock, average of mean bead size, percentage of beads with inter-bead distance less than 300 pixels, and percentage of beads with size lower than 100 pixels for anterior versus posterior part of the PVD neuron.


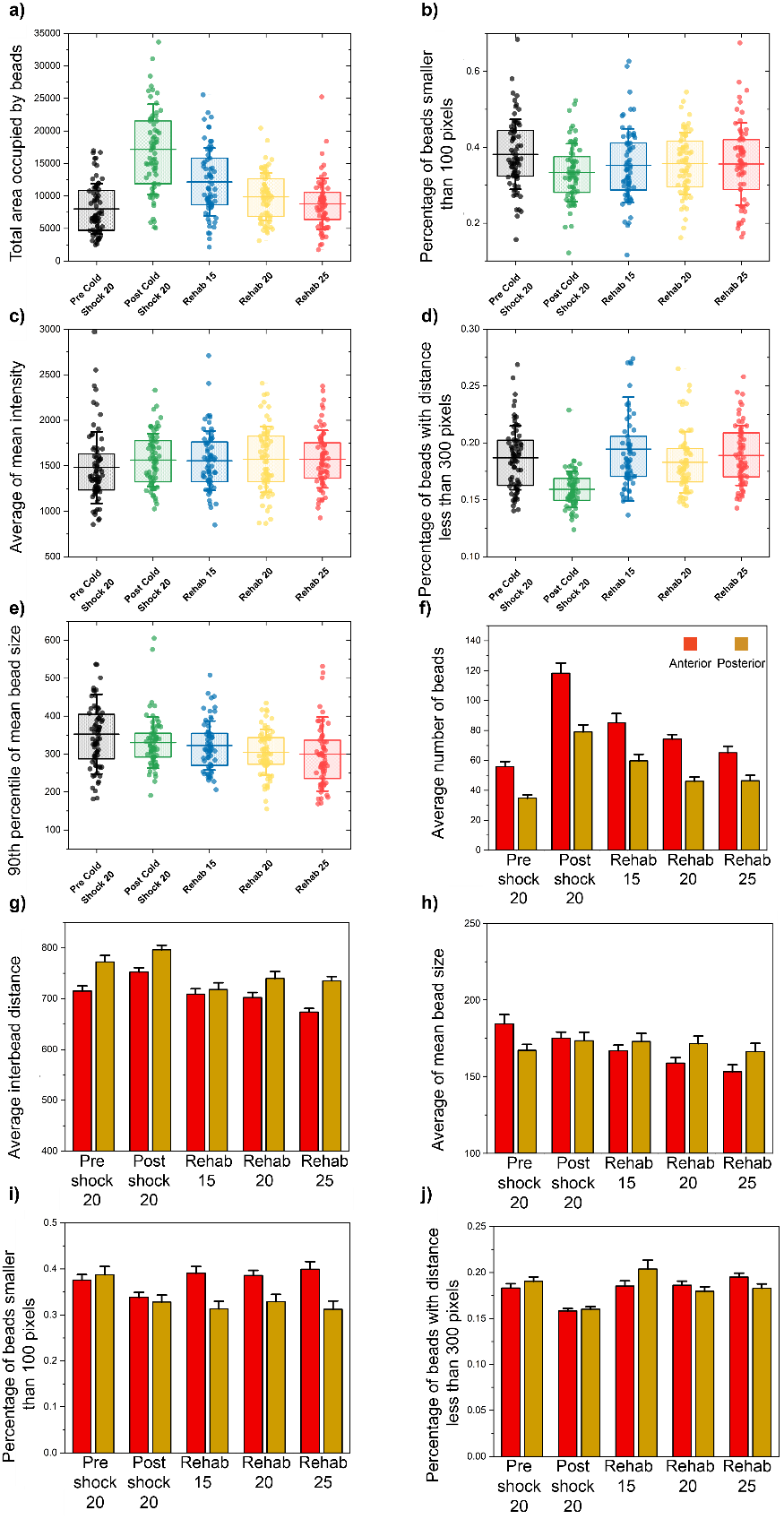


**Additional File 1: Figure S4.** PVD morphological changes caused by acute cold-shock is reversible and can be alleviated by post shock rehabilitation. **a)** Total area of PVD neuron covered with beads. **b)** Percentage of beads with size lower than 100 pixels. **c)** Average of the mean intensity of the beads. This line is a transcriptional line; thus, the change is the representative of the fluctuation in concentration on promoter along beads. **d)** The percentage of inter-bead distances lower than 300 pixels. **e)** 90^th^ percentile of beads fluorescence intensity. The lines are 25^th^ percentile, mean, and 75^th^ percentile. Whisker is the standard deviation. **f-j)** Average number of beads, average inter-bead distance of anterior versus posterior parts of PVD upon cold-shock, average of mean bead size, percentage of beads with inter-bead distance less than 300 pixels, and percentage of beads with size lower than 100 pixels for anterior versus posterior part of the PVD neuron.


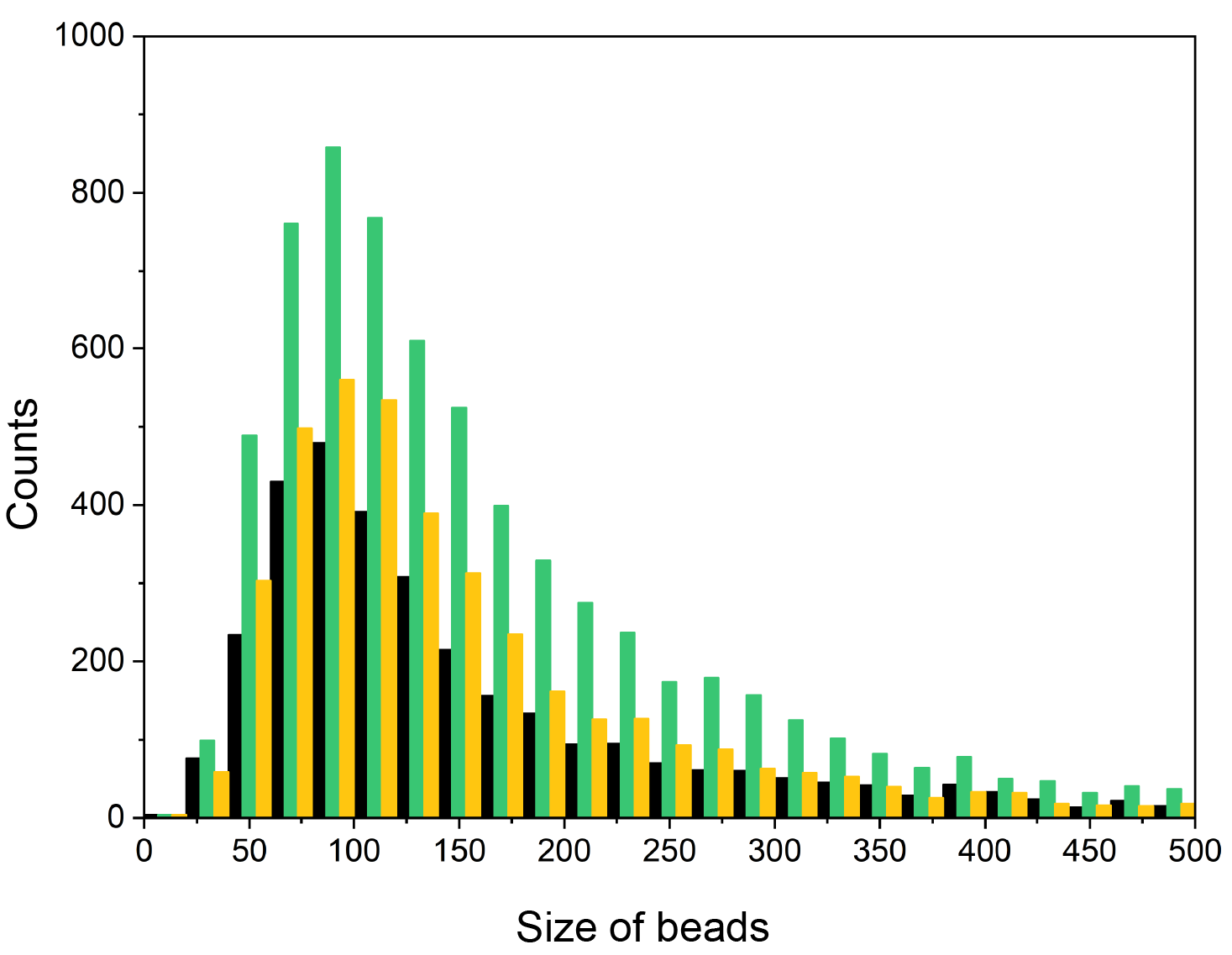


**Additional File 1: Figure S5.** Histogram distribution of individual bead size for recovery assay of a population cultured at 20 °C.


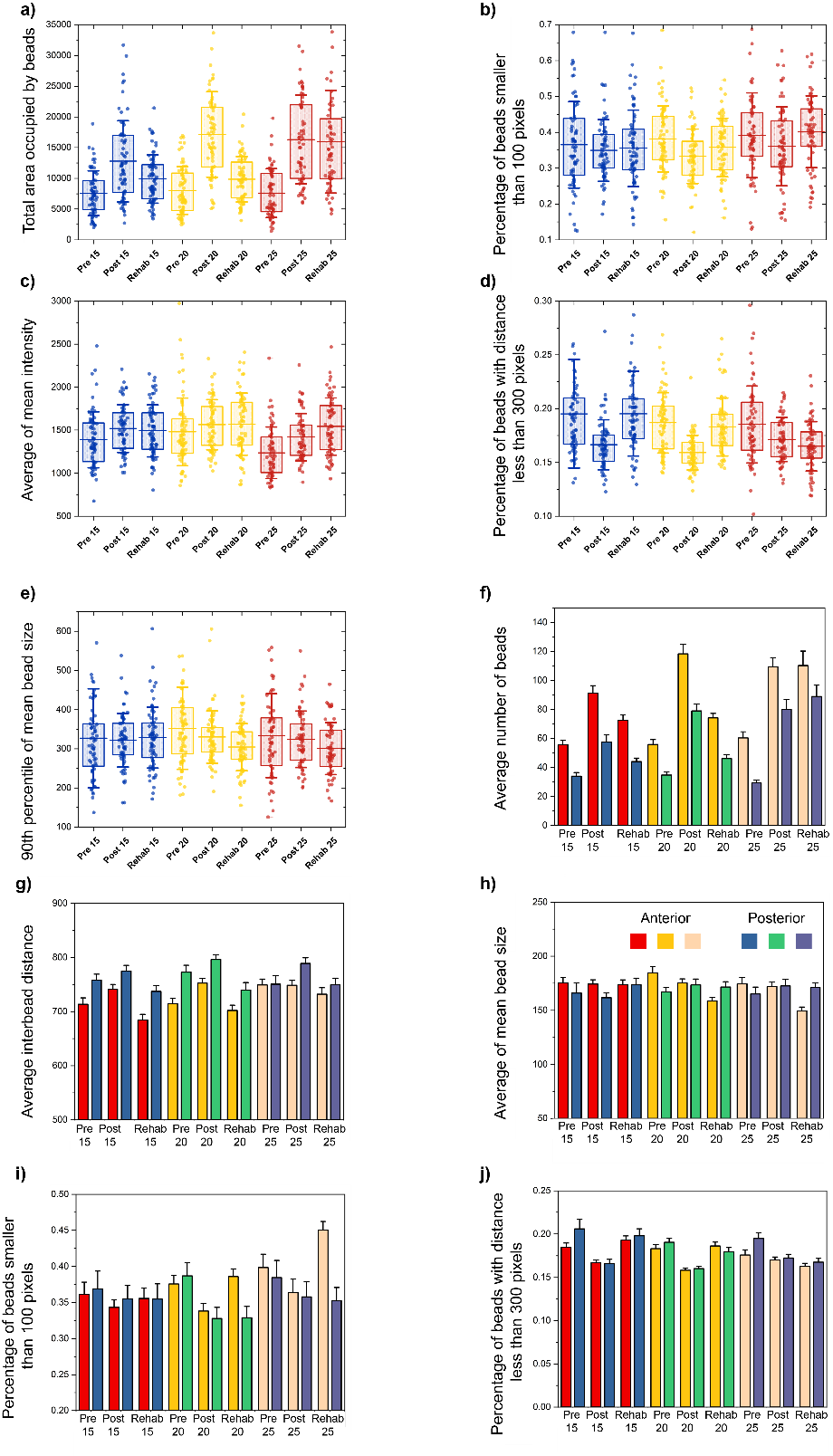


**Additional File 1: Figure S6.** Populations cultured at different temperature before being exposed to cold-shock respond in various ways to this external stressor. **a)** Total area of PVD neuron covered with beads. **b)** Percentage of beads with size lower than 100 pixels. **c)** Average of the mean intensity of the beads. This line is a transcriptional line; thus, the change is the representative of the fluctuation in concentration on promoter along beads. **d)** The percentage of inter-bead distances lower than 300 pixels. **e)** 90^th^ percentile of beads fluorescence intensity. The lines are 25^th^ percentile, mean, and 75^th^ percentile. Whisker is standard deviation. **f-j)** Average number of beads, average inter-bead distance of anterior versus posterior parts of PVD upon cold-shock, average of mean bead size, percentage of beads with inter-bead distance less than 300 pixels, and percentage of beads with size lower than 100 pixels for anterior versus posterior part of the PVD neuron.


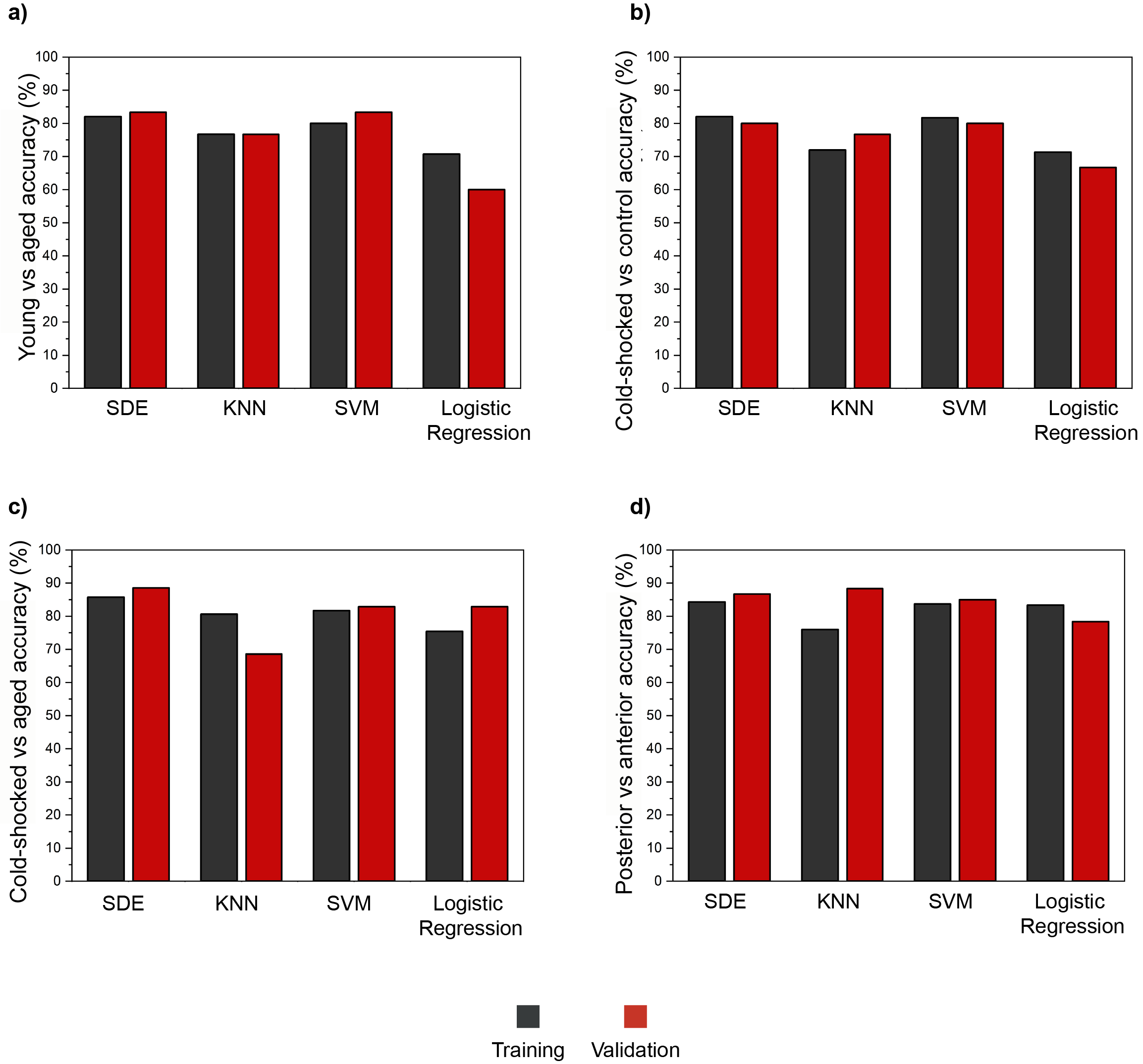


**Additional File 1: Figure S7.** Biological status of a nematode can be predicted based on PVD neuron’s health. **a)** Classification accuracy for young vs aged nematodes. The images were acquired from anterior part. **b)** Classification accuracy for cold-shocked vs control nematodes. The images were acquired from posterior part. **c)** Classification accuracy for cold-shocked vs aged nematodes. The images were acquired from posterior part. **d)** Classification accuracy for posterior vs anterior images of nematodes. N_T_ young= 50, N_T_ old= 100, N_V_ young= 10, N_V_ old= 20 for young vs aged classification. N_T_ cold-shocked= 75, N_T_ control= 75, N_V_ cold-shocked= 15, N_V_ control= 15 for cold-shock vs control classification. N_T_ cold-shocked= 75, N_T_ aged= 100, N_V_ cold-shocked= 15, N_V_ aged= 20 for cold-shocked vs aged classification. N_T_ anterior= 150, N_T_ posterior= 150, N_V_ anterior= 30, N_V_ posterior= 30 for anterior vs posterior classification.


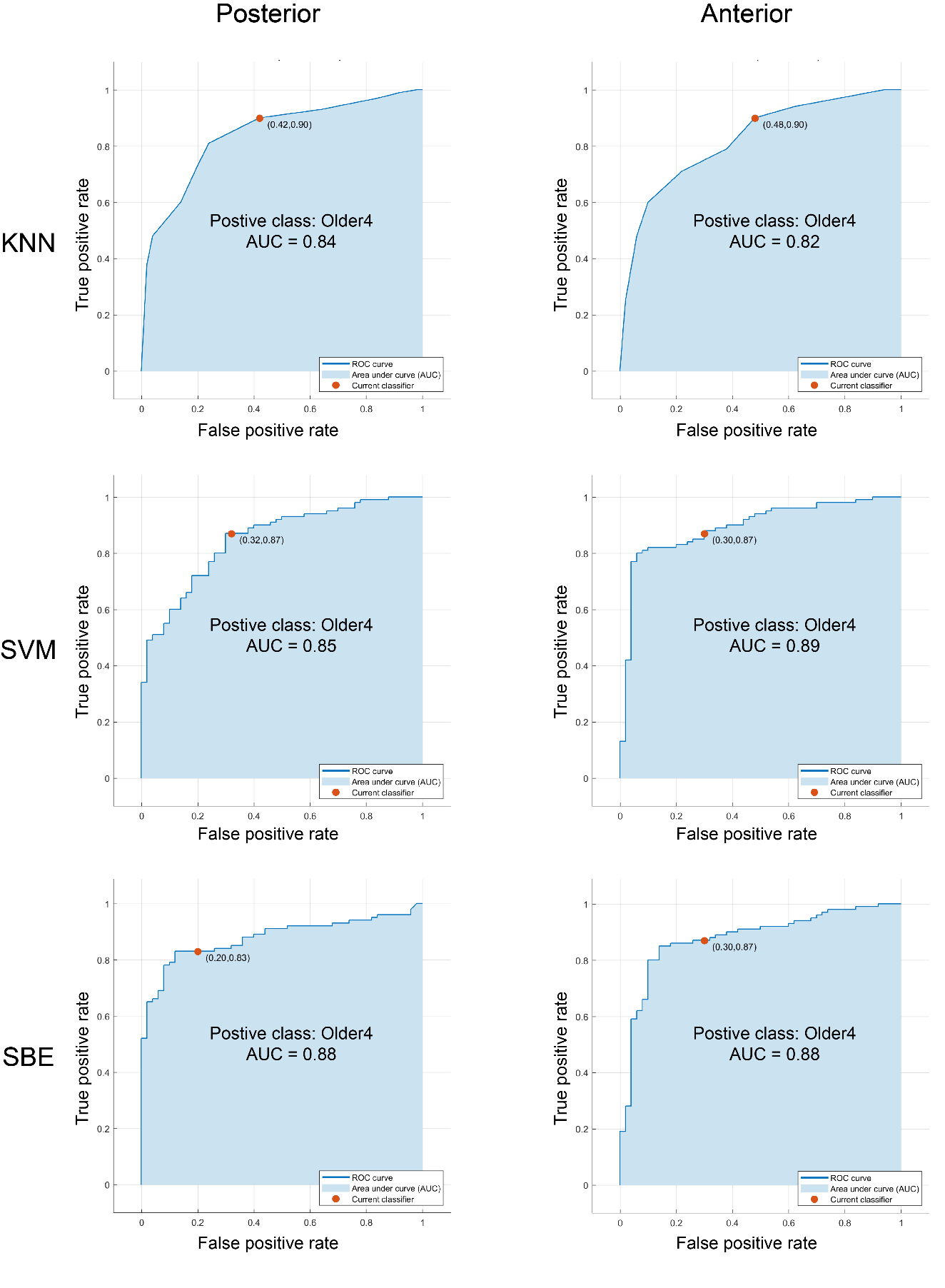


**Additional File 1: Figure S8.** The ROC curve for classification of young versus aged nematodes for anterior and posterior section of worms. The ROC curves of 3 classifiers (KNN, SVM, and SBE) have been plotted.


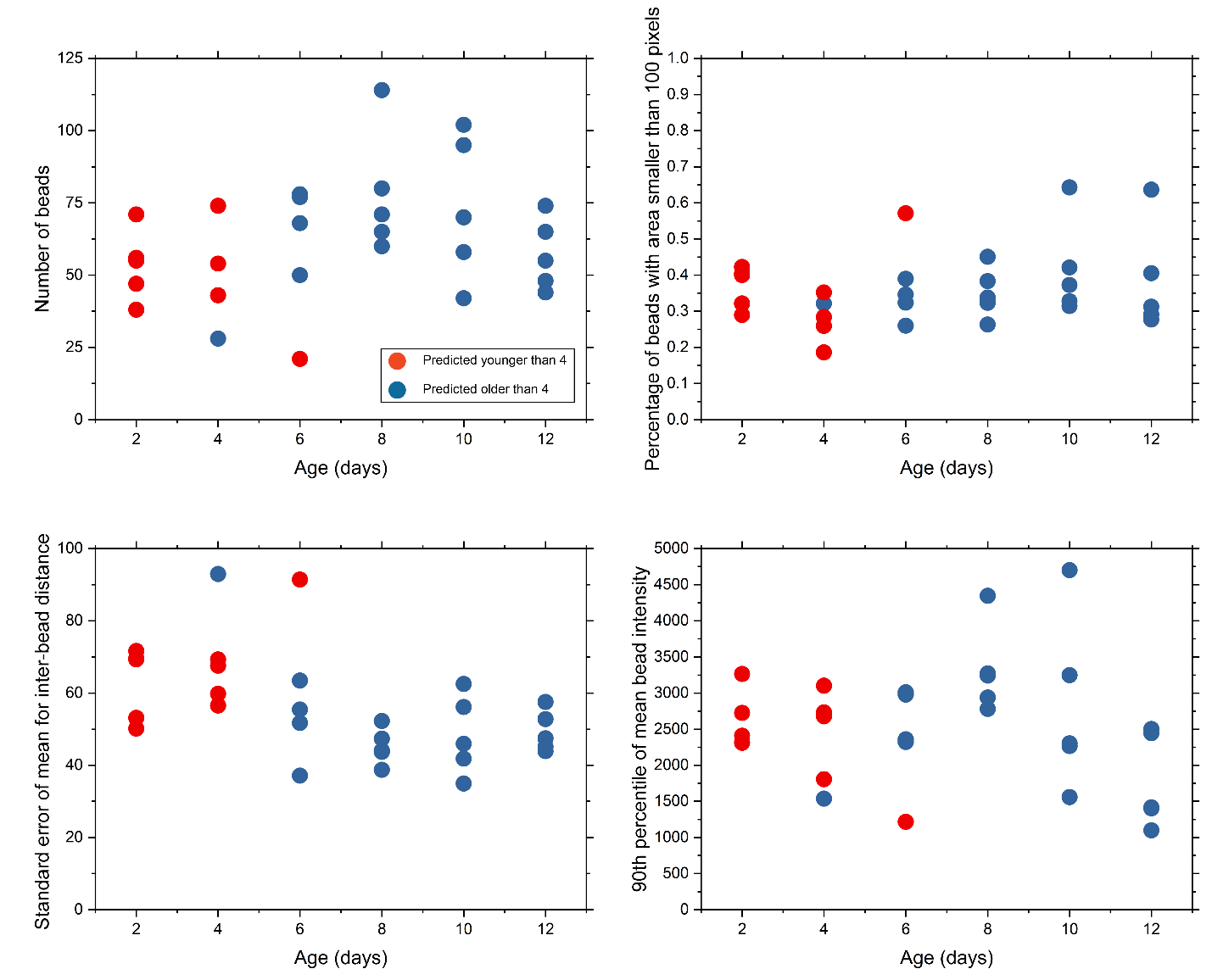


**Additional File 1: Figure S9.** The number of beads, percentage of beads with area smaller than 100 pixels, standard error of mean for inter-beads distance, and 90^th^ percentile of mean bead intensity plotted versus the age of nematodes used for validating the SBE classifier. The red color is prediction of classifier for nematodes to be younger than 4 days. The blue color is prediction of classifier for nematodes to be older than 4 days.

**Additional File 1: Figure S10.** Average of per-bead Jaccard index (intersection over union) for each image in the validation set, plotted vs the number of beads in each image. Error bars are standard deviation.
